# Zygoma implant under function: biomechanical principles clarified

**DOI:** 10.1186/s40729-023-00483-1

**Published:** 2023-06-22

**Authors:** Edmond Bedrossian, John Brunski, Bilal Al-Nawas, Peer W. Kämmerer

**Affiliations:** 1 Department of Oral and Maxillofacial Surgery, University of the Pacific, Dugoni School of Dentistry, 450 Sutter Street, Suite 2439, San Francisco, CA 94108 USA; 2 Stanford University School of Medicine, Phoenix, USA; 3 Department of Oral, Maxillofacial and Plastic Surgery, J. Gutenberg University of Mainz, Mainz, Germany; 4 University Medical Centre, Johannes Gutenberg University Mainz, Mainz, Germany

## Abstract

**Purpose:**

The purpose of this document is to clarify the biomechanical principles involved when zygoma implants are placed under functional loads.

**Methods:**

Two independent reviewers conducted electronic search of the literature from January 2000 to February 2023 describing the biomechanical principles involved using the zygoma implant for maxillary reconstruction. Articles describing the stresses within the zygoma implant, the maxillary bone and the zygoma bone under functional loads were included.

**Results:**

The lack of maxillary boney support at the implant platform resulted in significant higher stress measured within the zygoma implant as well as the zygoma bone.

**Conclusion:**

The maxilla is the primary support when zygoma implants are placed under functional loads. Quad-cortical stabilization of the zygoma implants and their cross-arch stabilization are recommended to reduce the degree of stress whenever possible.

## Background

**Fig. 1:**
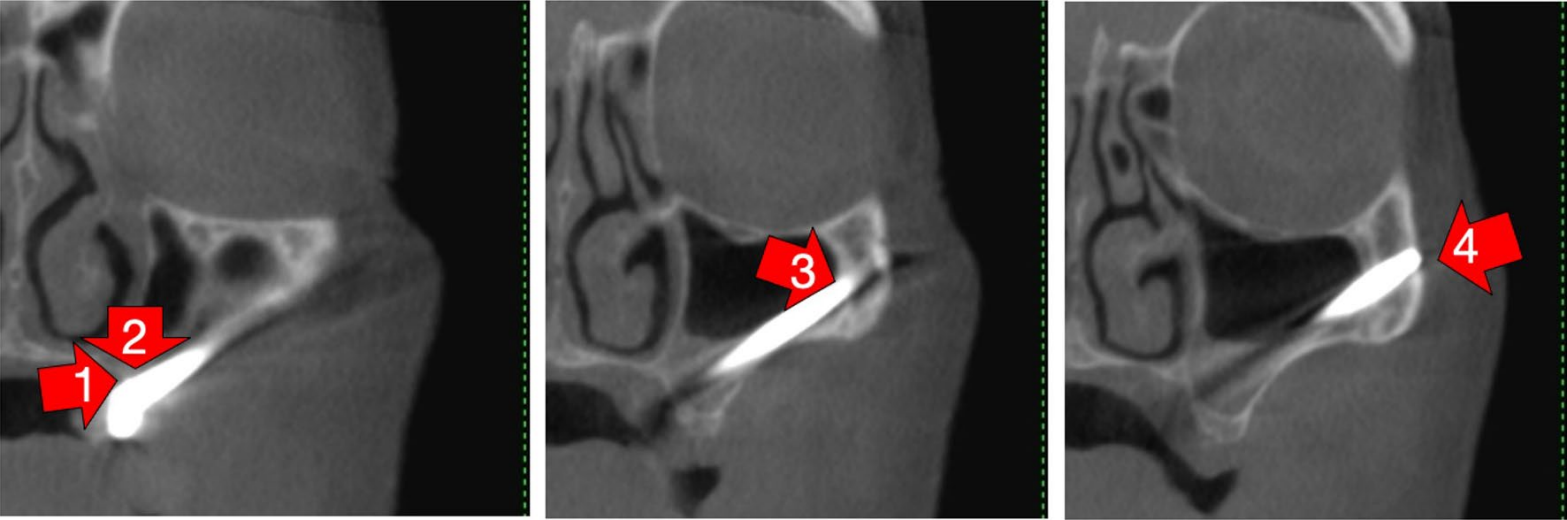
With the OST, the red arrows indicate the quad-cortically stabilized zygoma implant. 1. Lingual plate of maxillary alveolus; 2. floor of sinus; 3. roof of sinus; 4. lateral cortical plate of zygoma bone

The use of the zygoma bone, to establish posterior support for patients lacking maxillary boney volume was introduced by PI Branemark in 1988 [1]. The initial surgical protocol followed the delayed loading principles, which was later followed by the adoption of the immediate loading principles as introduced by Bedrossian and Chow in 2006 [2, 3]. The original surgical technique (OST) as described by Brånemark results in bi-cortical stabilization of the implant platform at the maxillary alveolus and bi-cortical stabilization of the apical portion of the implant within the body of the zygoma bone (Fig. 1).

Over the years, several modifications of the OST have been suggested as some authors have claimed unfavorable palatal emergence of the zygoma implant as well as associated maxillary sinus infections when using the OST. This document reviews the various modifications to the OST and encourages surgeons to be familiar with each proposed modification to evaluate whether the claims in reference to the OST are warranted. Also, understanding of the potential for compromising the biomechanical stability of the zygoma implant when modifying the OST should be considered before adopting any modifications of the original protocol.

### Original Branemark surgical technique

The original Branemark technique [4], OST, initiates the osteotomy through the cortical plate of the maxillary alveolar bone followed by penetration through the cortical floor of the maxillary sinus. The trajectory of the drill may be inside or outside the maxillary sinus depending on the contour of the lateral wall of the maxillary sinus as it approaches the zygoma bone. The drill then enters the base of the zygoma bone, travels through the body of the zygoma bone and eventually penetrates out through its lateral cortical wall. The two maxillary cortical plates provide bi-cortical stabilization of the implant platform. The additional two cortical plates at the zygoma bone provide the second bi-cortical stabilization of the apical portion of the zygoma implant. Thus, the zygoma implant is *quad-cortically stabilized*. As mentioned earlier, criticism of the OST have included, an unfavorable emergence of the implant platform described as being “too palatal” as well as the potential for sinus infections. It is therefore prudent to discuss these claims in detail to better understand the OST.

#### Palatal emergence

The two common mistakes made when initiating the osteotomy for the placement of zygoma implants is to be “too anterior” or “too palatal” due to the relative difficult access and or the disorientation of the inexperienced surgeon. If the osteotomy is initiated on the palatal process of the maxilla instead of the palatal/lingual wall of the maxillary alveolus, the incorrect osteotomy may result in the unfavorable palatal emergence of the zygoma implant with potential for biological and prosthetic complications. It is critical to appreciate that the proper execution of the OST will not result in the zygoma implant platform to be in the palate.

#### Maxillary sinus infections

Over the years, some clinicians have alluded that the presence of titanium within the maxillary sinus may cause an inflammatory process or infections. To address these concerns, Branemark studied the reaction of the Schneiderian membrane to the presence of titanium implant with in the maxillary sinus. He collaborated with head and neck surgeon/otolaryngologist, Bjorn Petruson to study the reaction of the Schneiderian membrane to the presence of titanium in patients treated with the zygoma implant. In 2004, the results of this study [5] showed the absence of inflammation in the surrounding mucosa of the sinus when examined directly using a trans-nasal endoscope. Their observation also revealed that in some cases the zygoma implant inside the maxillary sinus may be covered by the Schneiderian membrane, while in others, there was only partial coverage of the zygoma implant by the membrane. Petruson also emphasized that there was no infection or increased secretions of the Schneiderian membrane which could be disrupted during the preparation of the osteotomy or during the placement of the zygoma implant. It has been the understanding of the authors that most clinicians experienced in the placement of the zygoma implants using the OST, support the findings of Branemark and Petruson.

Modifications of the OST have been suggested to include the preparation of the osteotomy to place the mid-portion of the implant outside the maxillary sinus. Review of these techniques and contrasting them with the OST is important to understand the potential risks and benefits of each approach.

### Extra-sinus technique

**Fig. 2:**
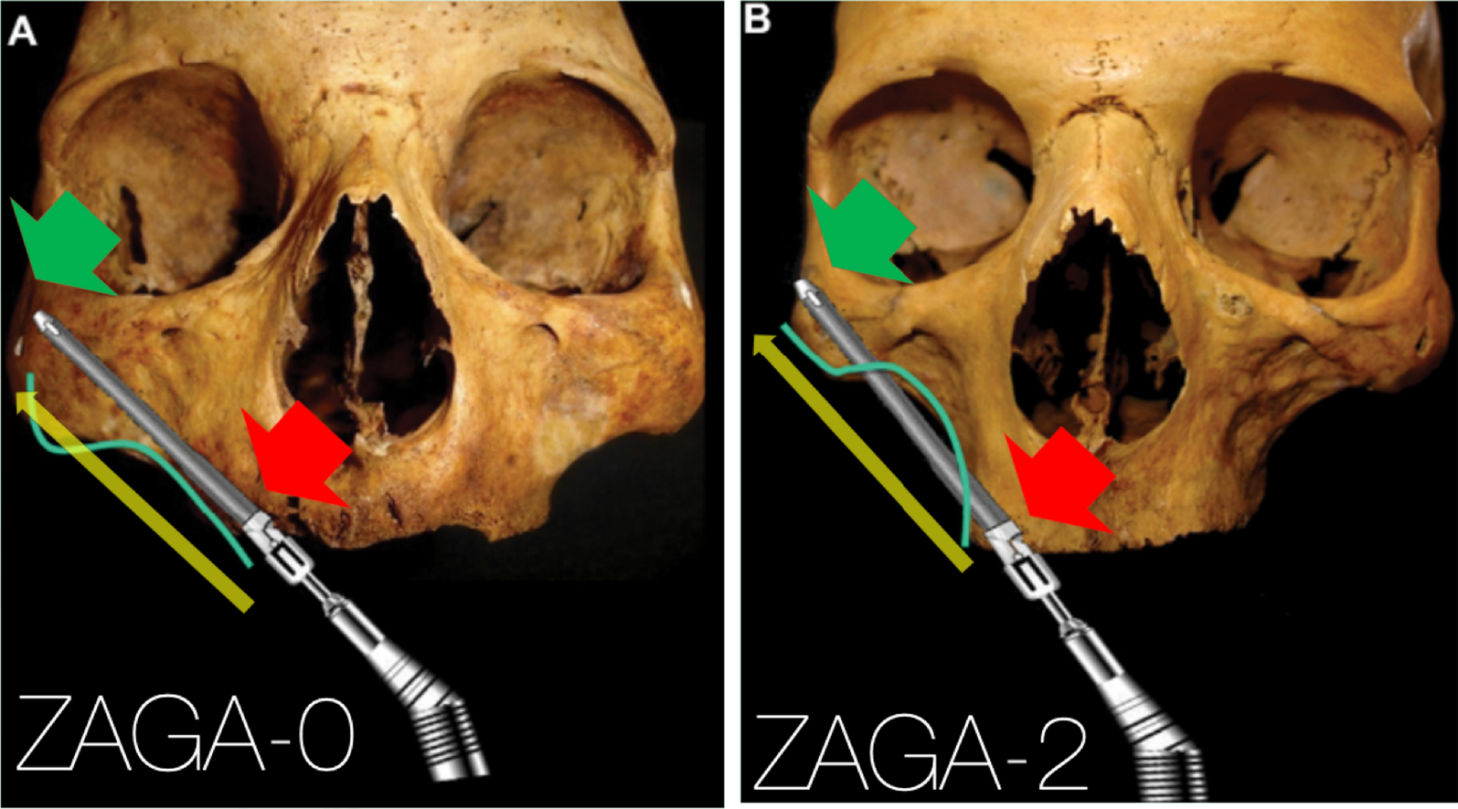
**a**, **b** The starting point and the end point are represented by the red and green arrows, respectively. The yellow arrow represents the implant trajectory which is exactly the same in **a** representing the OST and **b** representing the “extra-sinus” technique; from Kato et al.

The “exteriorized” approach or later often called the “extra-sinus” approach was described as a technique without making a maxillary antrostomy. From an anatomical viewpoint, the starting and the end point and hence the trajectory of the zygoma implants placed following the “extra-sinus” approach is exactly the same as the OST (Fig. 2). When studying this technique, surgeons must appreciate that the “extra-sinus” position of the “mid portion” of the zygoma implant is a consequence of the concave anatomy of the patient’s lateral maxillary sinus wall and not a consequence of a so called “new technique”. Therefore, the “extra-sinus” approach is not a different technique, rather, it is an observation of the outcome of the implant trajectory based on the patient’s lateral sinus wall anatomy.

In 2008 [6], the “extra-sinus” technique was further modified by Malo. The modification was the removal of the maxillary alveolar bone, eliminating the ability to have bi-cortical anchorage of the zygoma implant platform. The surgical procedure was made easier by this modification; however, the biomechanical changes resulting in a long-cantilevered implant anchored only at the zygoma bone was not taken into consideration. Therefore, this modification raised many questions as to the difference between a quad-cortically stabilized zygoma implant as described by the OST and the bicortically stabilized zygoma implant as described by the extra-sinus technique.

In attempt to answer these questions, this document reviews the biomechanical principles involved when the zygoma implant is placed under functional loads. By comparing and contrasting the difference between the bi-cortical vs quad-cortically anchored zygoma implants, the implant teams can adopt the most scientifically favorable surgical and prosthetic protocols when treating their patients using the zygoma implant.

### Surgical and prosthetic biomechanical principles

**Fig. 3:**
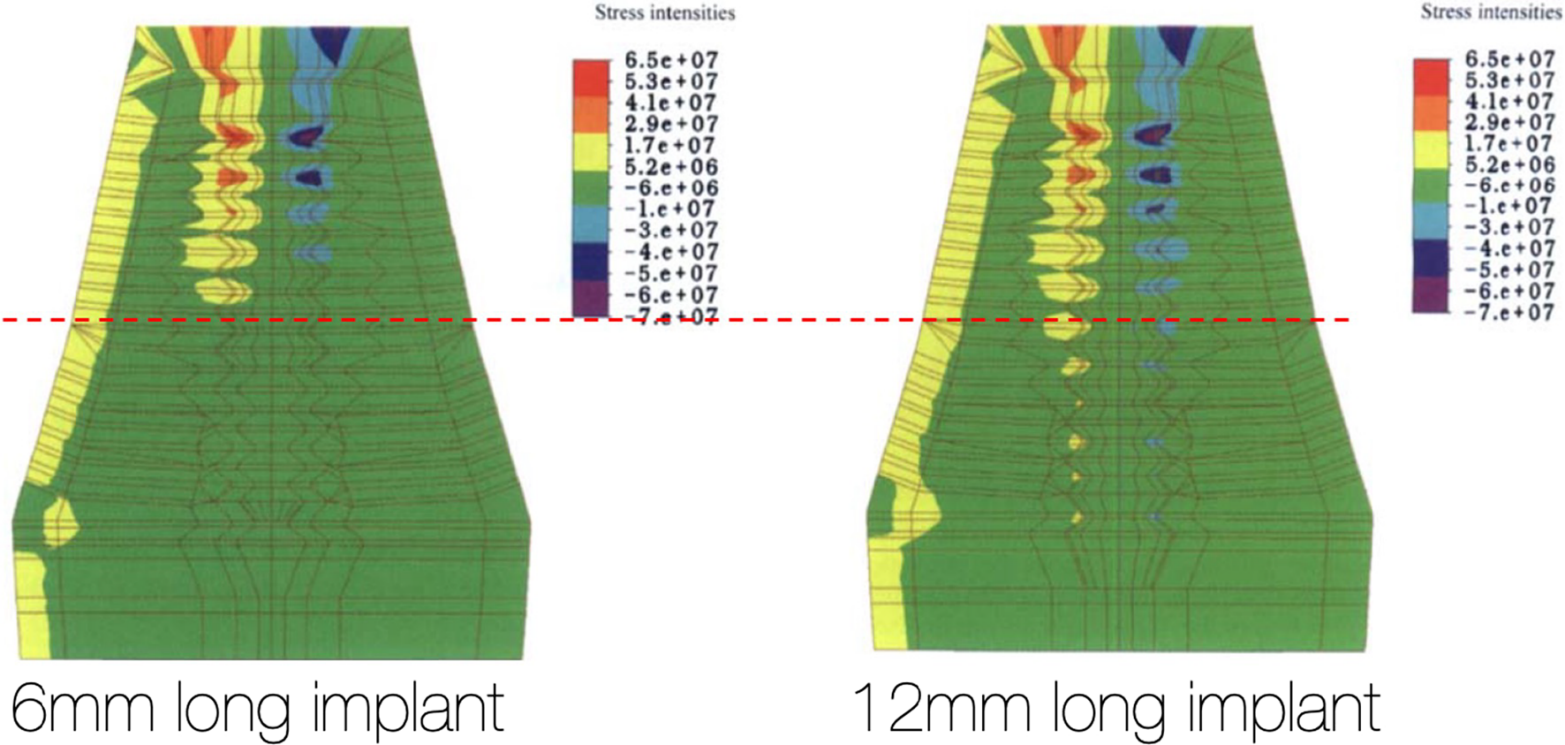
The red-dotted line shows the concentration of loads to be the same in 6-mm-long implant as well as a 12-mm-long implant

The loads applied to dental implants supporting a fixed prosthesis during mastication are, centric occlusion (vertical forces) and lateral excursions (Horizontal forces). As described by Ranouard et al., centric and lateral occlusal loads are concentrated at the implant platform and the first 5 mm of the implants length regardless of the implant length [7–11] (Fig. 3).

In 2002, Iplikcioglu and Akca also reported that the increase in the length of the implant does not decrease the stress levels within the implant platform [12] with the stresses always concentrated at the implant platform. Nishihara [13], Hedia [14] and Rangert [15, 16] also supported the findings of previous authors confirming that the highest stresses are seen at the crestal cortical bone around the implant platform in CO and at the first 3–5 mm of the implant length in lateral excursions regardless of the implant length.

As the zygoma implant is a unique implant with the following distinguishing features:

- It is a long implant generally ranging from 30 to 60 mm.
- It is placed in a non-axial trajectory.
- It may be anchored by the maxilla and the zygoma (quad-cortically stabilized), or stabilized only by the zygoma bone (bicortically stabilized).

The application of the general biomechanical principles of the “standard” implant as described by Ranouard and others may raise questions among different implant teams. Therefore, it is important to revisit the various reports in the existing body of published literature to clarify whether it is the *maxillary alveolar bone or the zygoma bone* responsible as the primary support of functional loads applied in centric as well as lateral excursions.

### Transferring of functional loads: the surgical perspective

Kato, Ujigawa, and Freedman [17–20] have attempted to describe the transfer of functional loads to the zygoma implant, the maxillary alveolar bone as well as the zygoma bone in their finite Element Analysis (FEA). Forces transferred to prosthesis supported by zygoma implants have also been studied comparing “lone-standing zygoma implants” to “cross-arch splinted zygoma implants” under functional loads.

**Fig. 4:**
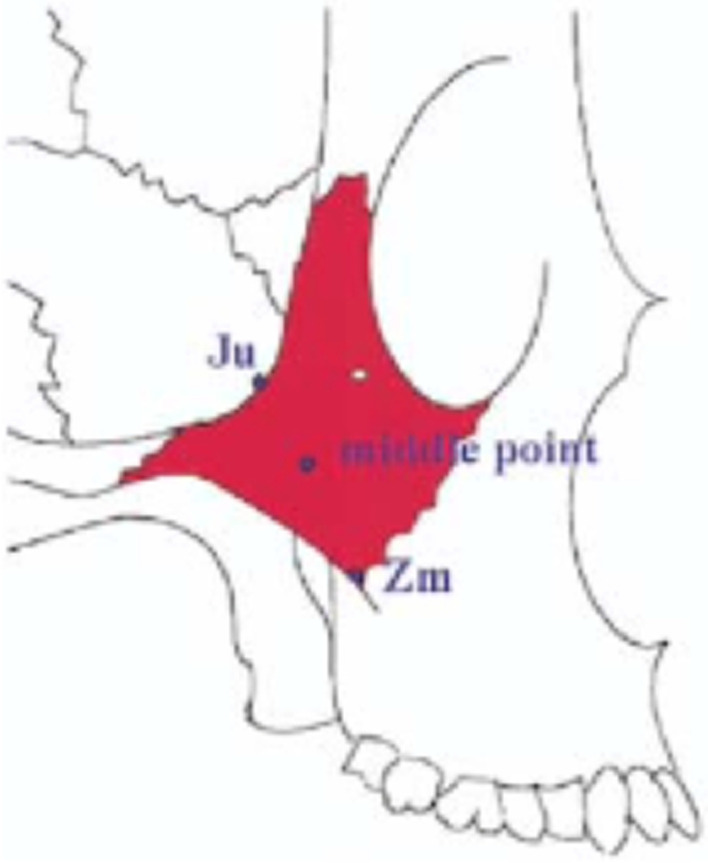
Point “JU” has the densest boney topography

Kato in 2005 using micro-CT scans of the zygoma bone isolated from dry skulls, described the trabecular density of 3 different points on the body of the zygoma bone (Fig. 4).

Point “JU” was determined to have the densest trabecular pattern. Citing the effect of muscle pull on long bones from the orthopedic literature, Kato concluded that the zygoma bone supports the loads applied to zygoma implants due to density of bone at frontozygomatic notch (point JU). However, in critical review of the anatomy, there are no major muscles which originates or inserts on the body of the zygoma bone. The lateral surface of the zygoma bone is the origin of the zygomaticus major and minor muscles (muscles of facial expression) which blend and insert into the muscles of the upper lip. The contraction of these small muscles is unlikely to contribute to increased bone density of the zygoma bone. Surgeons should also appreciate that the apical portion of the zygoma implant is in the body of the zygoma bone referred to as “middle point” by Kato and not at the “JU” point. In light of the critical analysis of Kato’s description and study of the zygoma anatomy, Kato’s conclusions which state that the occlusal forces are borne only by the zygomatic bone should be questioned.

**Fig. 5:**
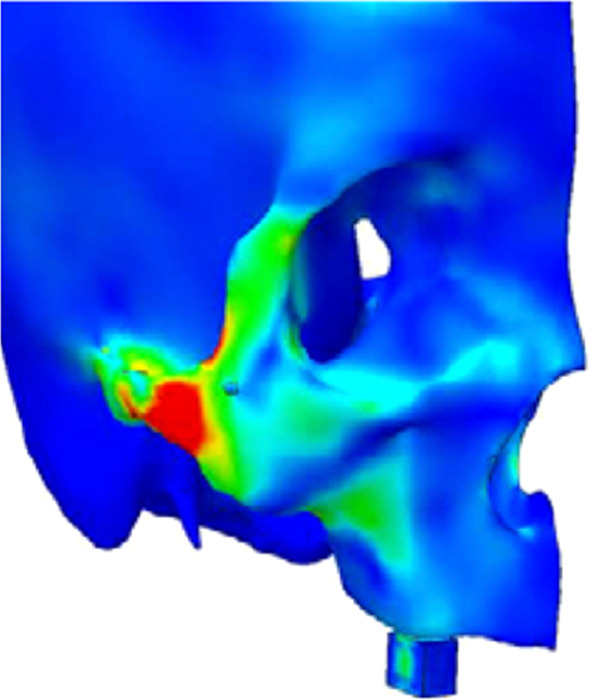
The illumination of the masseter muscle on the zygomatic arch during centric occlusion as described by Ujigawa

Ujigawa et al. in 2007 also described the “lighting of the zygomatic arch” in their FEA, citing that the zygoma bone is the primary support when the zygoma implant is placed under functional loads. Critical review of his article and the illustration from his FEA, Fig. 5, once again shows that it is indeed the masseter muscle which is activated under function (the highlighted red zone) and not the zygoma bone. Therefore, like Kato’s report, the conclusion reached in this article in regard to the zygoma bone being the primary support of the zygoma implant under function is once again not scientifically or anatomically supported.

**Fig. 6:**
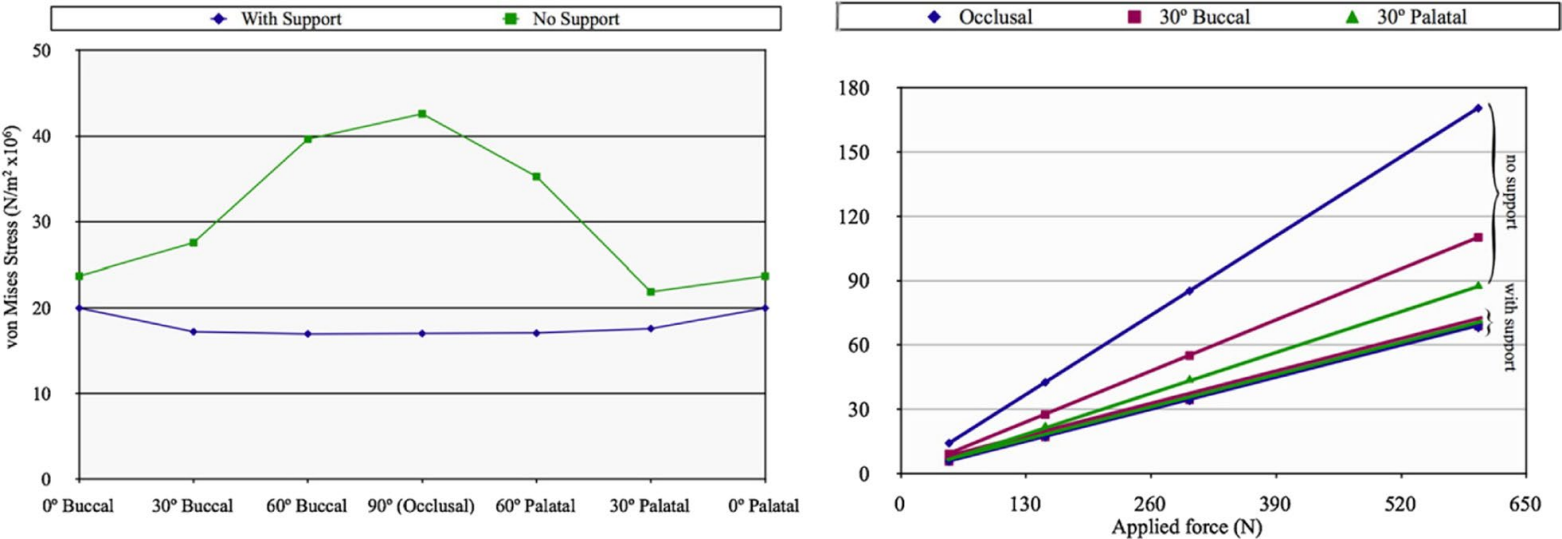
Increased loads at the zygoma implant platform in absence of maxillary crestal boney support

Freedman in 2013, also described the loads applied to the zygoma implant under function. In his FEA, two identical models both with the zygoma implants stabilized at the implant platform by the maxilla as well as stabilized at their apical portion by the zygoma bone was created. One model was then modified by deliberately removing the crestal bone which was stabilizing the “zygoma implant platform”. The second model was kept intact with stabilization at the implant platform as well as stabilization of the apex of the implant. Both models were placed under centric as well as lateral loads. His data clearly demonstrated increased loads at the implant platform in the model which did not have maxillary alveolar boney stabilization (Fig. 6).

**Fig. 7:**
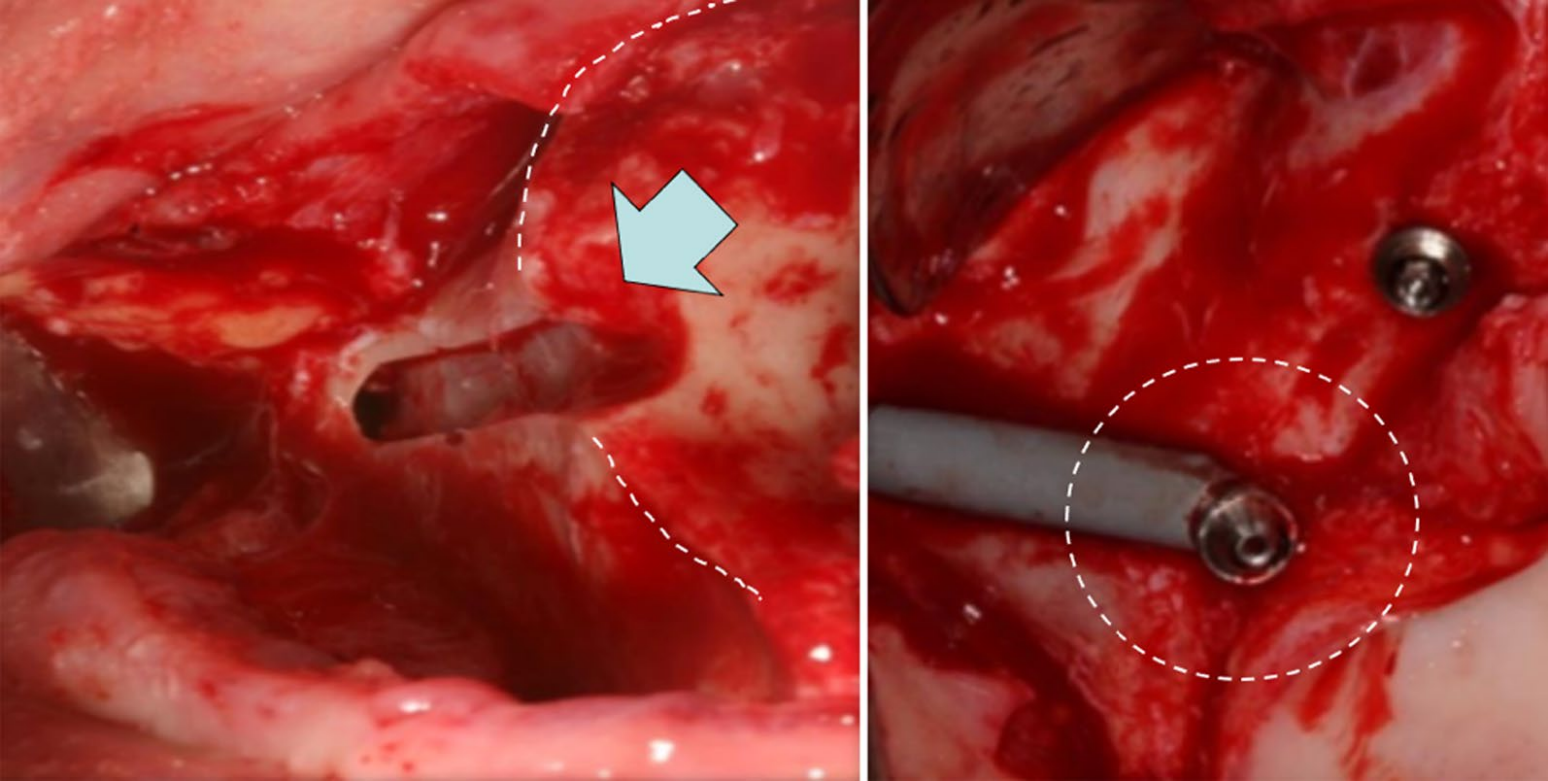
The arrow points to the intentional removal of the maxillary crestal bone. The implant platform is not supported by the maxillary alveolus

In 2015, Freedman in a new FEA studied the “extra-sinus technique” where the alveolar bone is intentionally removed for the so claimed “more prosthetically appropriate” position of the zygoma implant platform (Fig. 7).

**Fig. 8:**
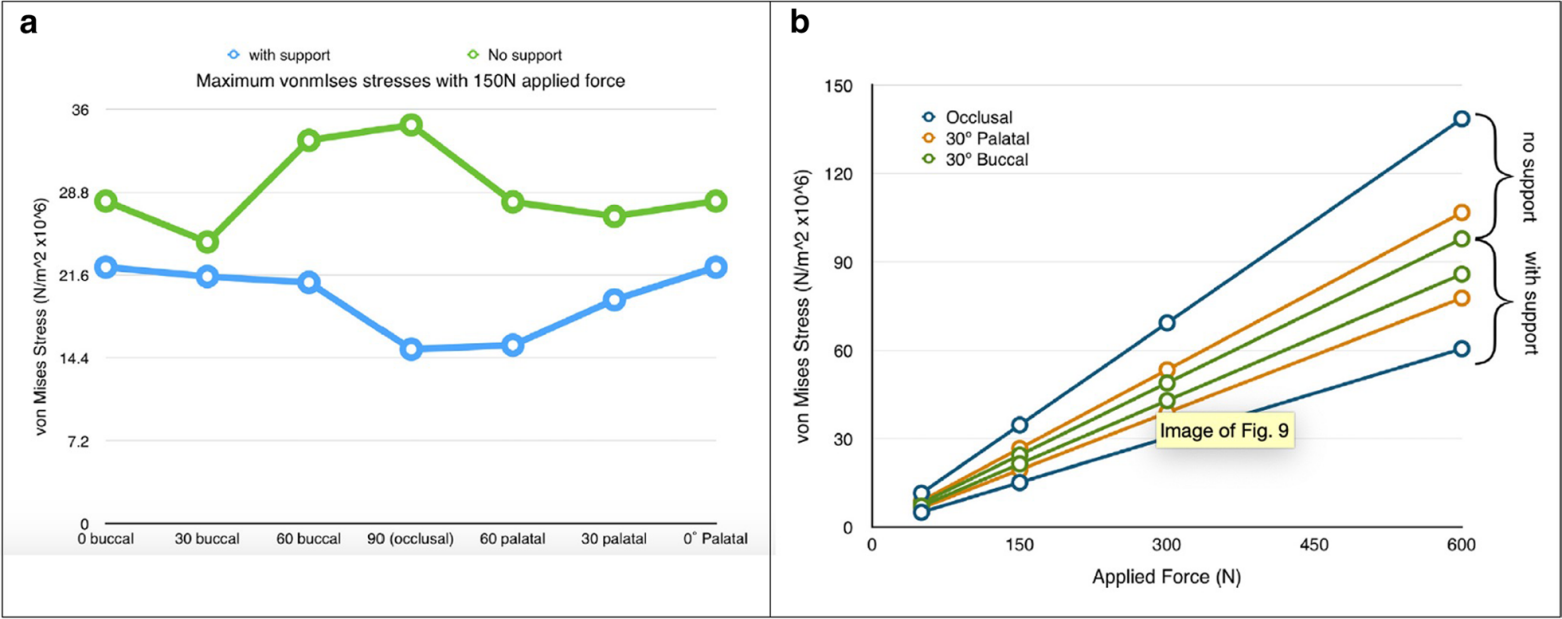
**a**, **b** Increased loads in centric occlusion as well as in lateral excursion without maxillary alveolar support

Once again, the results of the FEA were a significant increase in the levels of stress at the zygoma implant platform in the model with no boney support at the maxillary alveolar bone (Fig. 8a, b).

The 2013 and the 2015 studies by Freedman clarified that the stabilization of the zygoma implant platform at the residual maxillary crest is critical for appropriate force distribution.

### Transferring of functional loads: the prosthetic perspective

**Fig. 9:**
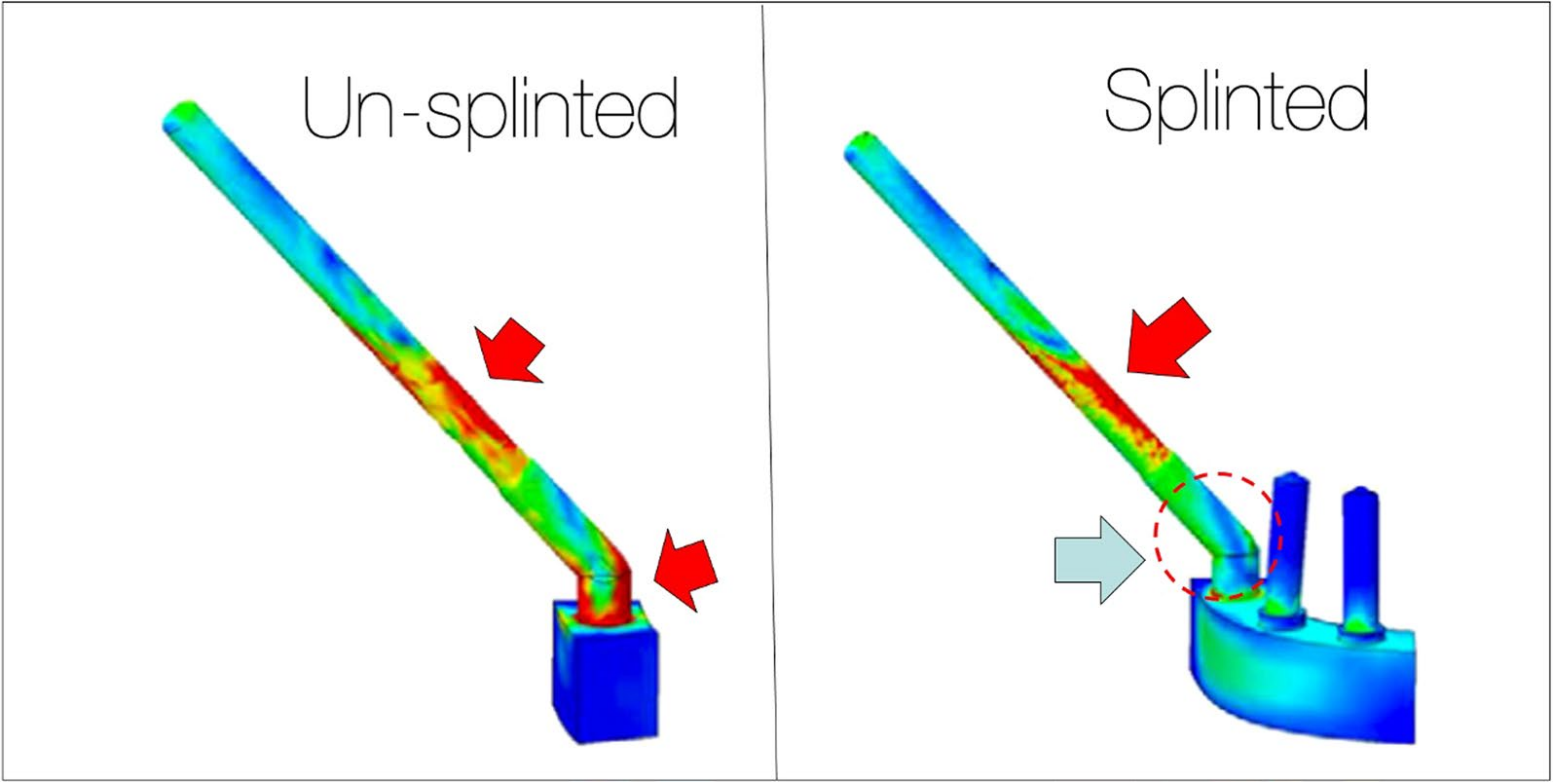
Ujigawa; arrows indicate the stress within the zygoma implant. Significant reduction of occlusal loads, grey arrow, when the zygoma implant is cross-arch splinted

Ujigawa et al. [19] in their study also specifically described the occlusal stresses applied to the full arch fixed prosthesis supported by zygoma implants. Two models were created in this FEA. The first model with posterior zygoma implants splinted with the anterior axial implants and a second model with the zygoma implants un-splinted. Ujigawa confirmed the work of Renouard and others that the occlusal and lateral loads are borne at the zygoma implant platform and the first 5 mm of the zygoma implant. Therefore, splinting the zygoma implants reduces the degree of stress at the implant platform (Fig. 9) and the loading of un-splinted zygoma implants are not recommended.

To further study and evaluate the stress in the zygoma implant, the maxillary bone as well as the zygoma bone, the Bedrossian–Brunski models were created in 2023.

### Bedrossian–Brunski model

**Fig. 10:**
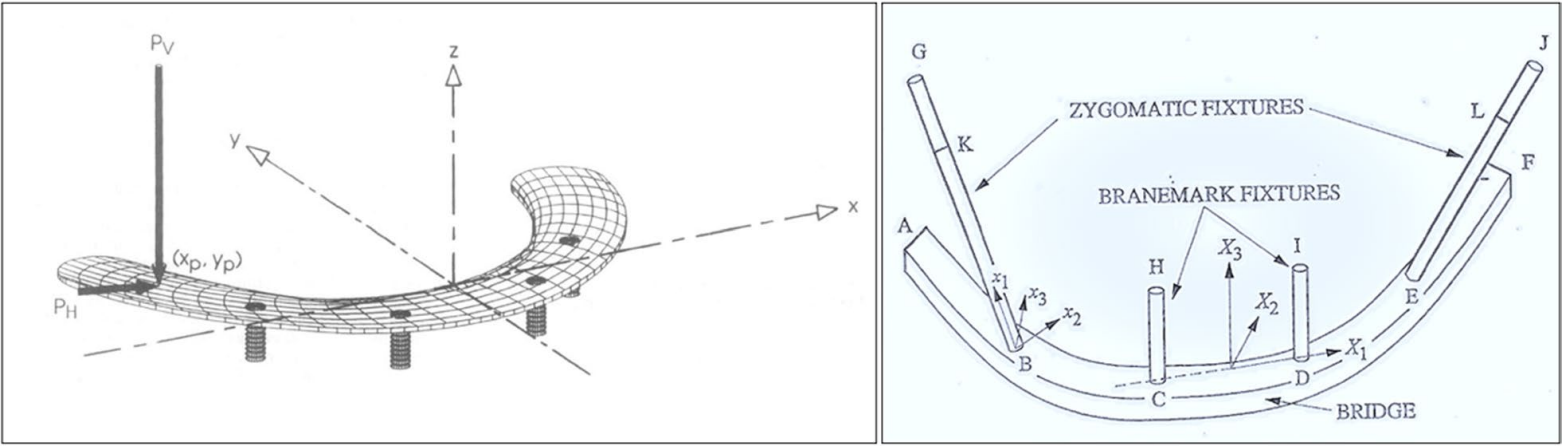
The Skalak and the Morgan and James model

To verify and update the contemporary literature in regard to the surgical as well as the prosthetic biomechanical principles using the zygoma implant, Bedrossian and Brunski modified the Skalak as well as the Morgan and James models (Fig. 10) [21–23].

**Fig. 11:**
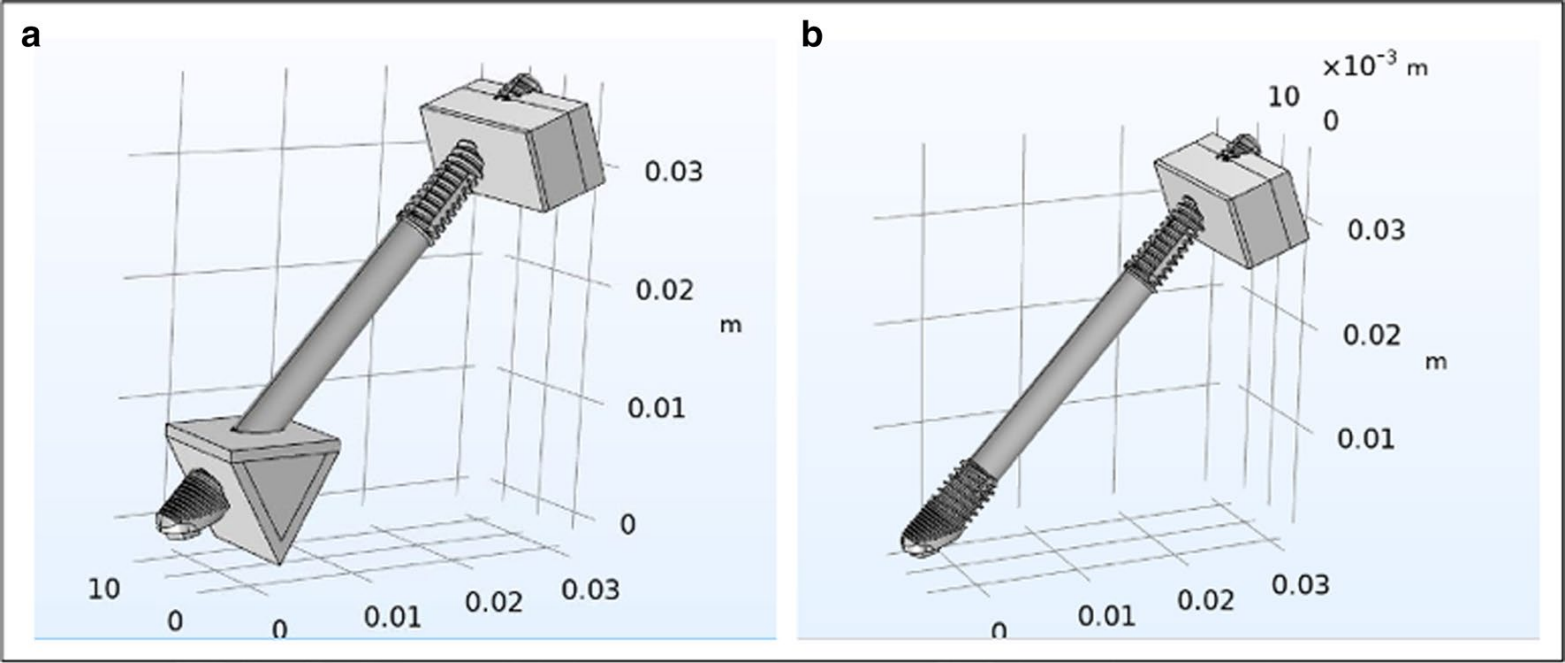
**a**, **b** The quad-cortical (BBQ) and bicortically (BBB) stabilized surgical models

The modifications included the removal of the posterior cantilevers, zygoma implants simulating the posterior support and two axial implants in the anterior with a cross-arch splinted bar. Two versions of boney support for the zygoma implants were created. One zygoma model had boney supports at the zygoma implant platform and its apex; this is referred to as the Bedrossian–Brunski quad-cortical model (BBQ); the second model had the zygoma implant stabilized only at its apical portion in the zygoma bone; the Bedrossian–Brunski bi-cortical model (BBB) (Fig. 11a, b).

**Fig. 12:**
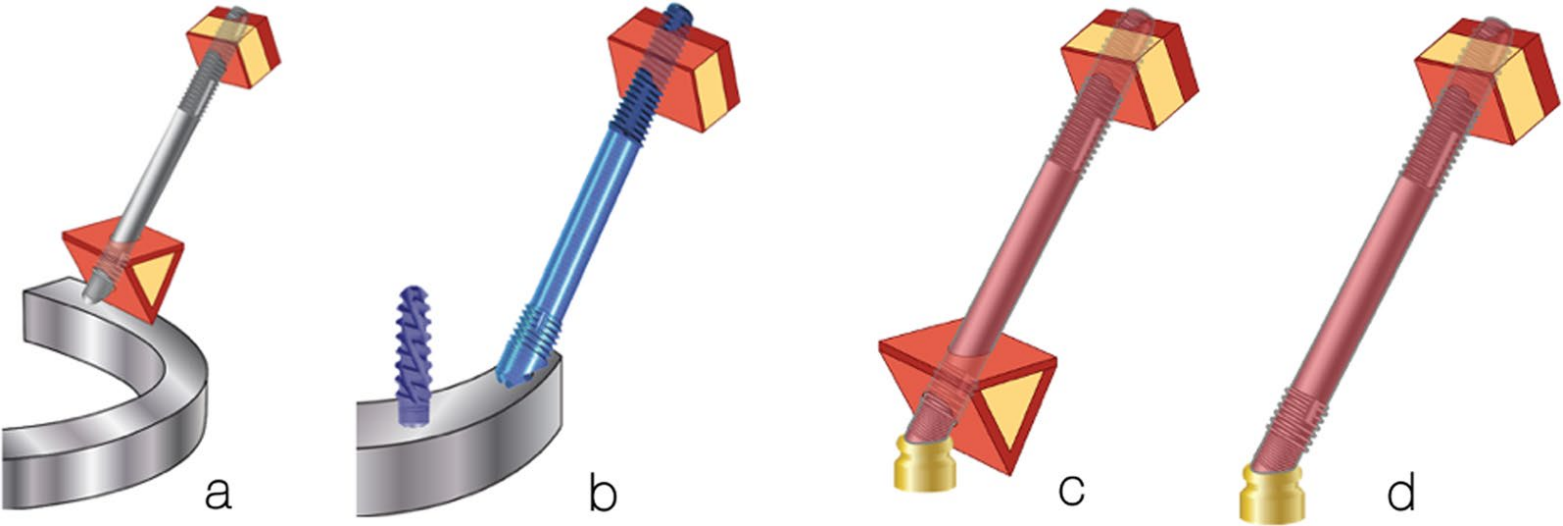
**a**–**d** Cross-arch splinted BBQ and BBB models (**a**, **b**) and the lone-standing BBQ and BBB models (**c**, **d**)

To study the effect of functional loads on prosthesis supported by zygoma implants, cross-arch splinted BBQ and BBB zygoma implant models vs lone-standing BBQ and BBB zygoma implants were created (Fig. 12a–d).

### Surgical models

**Fig. 13:**
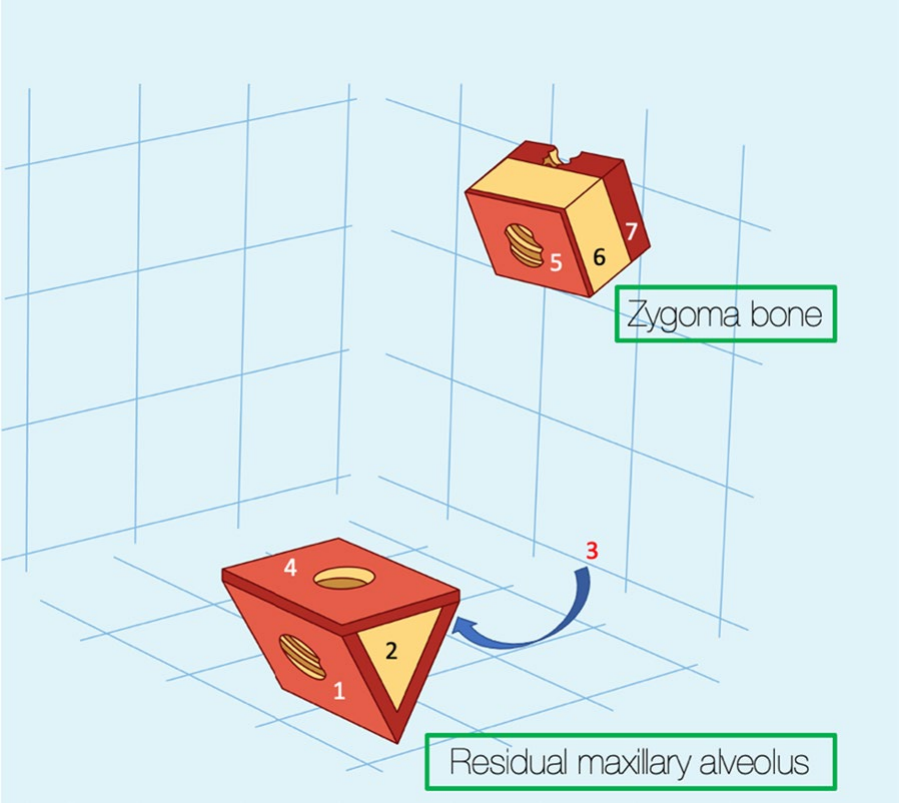
1, is the cortical lingual wall of the residual maxillary alveolus. 2, the cancellous portion of the residual maxillary alveolus. 3, the cortical buccal wall of the residual maxillary alveolus. 4, the cortical floor of the maxillary sinus. 5, the cortical wall of the base of the zygoma bone (roof of the maxillary sinus).6, the cancellous bone of the body of the zygoma bone. 7, the cortical outer cortex of the zygoma bone

To simulate the residual maxillary bone, a 6 mm-by-6 mm triangular illustration was created simulating the maxillary alveolus. The body of the zygoma bone is represented by the 6 mm-by-6 mm rectangular illustration simulating the zygoma bone. The cortical–cancellous topography of the maxillary ridge and the zygoma bone is represented in Fig. 13.

#### Quad-cortical stabilization

**Fig. 14:**
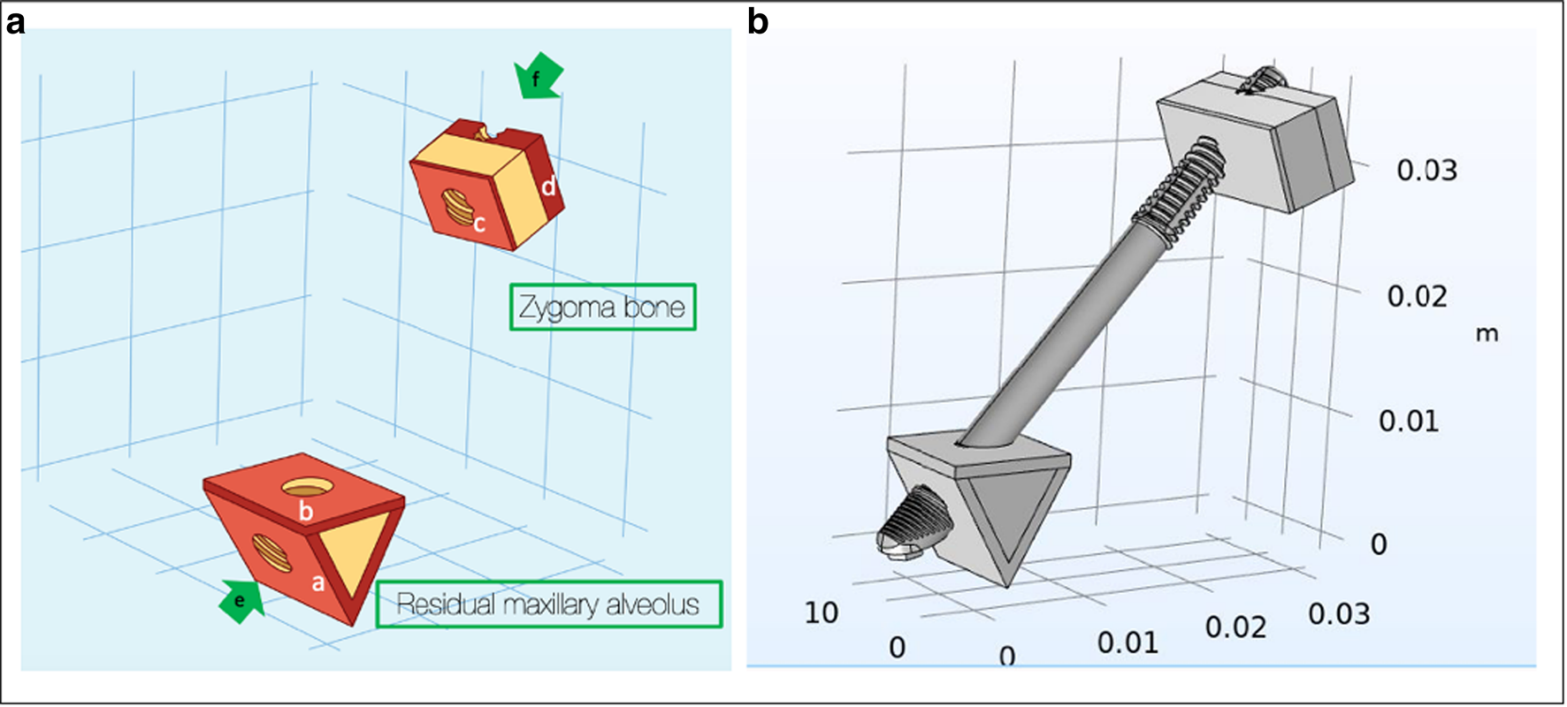
**a**, **b** Trajectory and the quad-cortical points for stabilizing the zygoma implant

This refers to the stabilization of the zygoma implant at its platform as well as its apical portion. The trajectory of the zygoma implant begins at point “e”, through the maxillary alveolus in a superior-lateral and slightly anterior direction, entering the base of the zygoma bone and exiting at point “f”. Therefore, the zygoma implant platform is stabilized by the cortical bone of the lingual wall of the maxillary residual ridge and the cortical floor of the maxillary sinus, a and b, respectively. The apex of the implant is once again stabilized by the cortical bones of the roof of the maxillary sinus as well as the lateral cortical wall of the zygoma bone, c and d, respectively (Fig. 14a, b). Hence, this implant has quad-cortical stabilization.

#### Bi-cortical stabilization

**Fig. 15:**
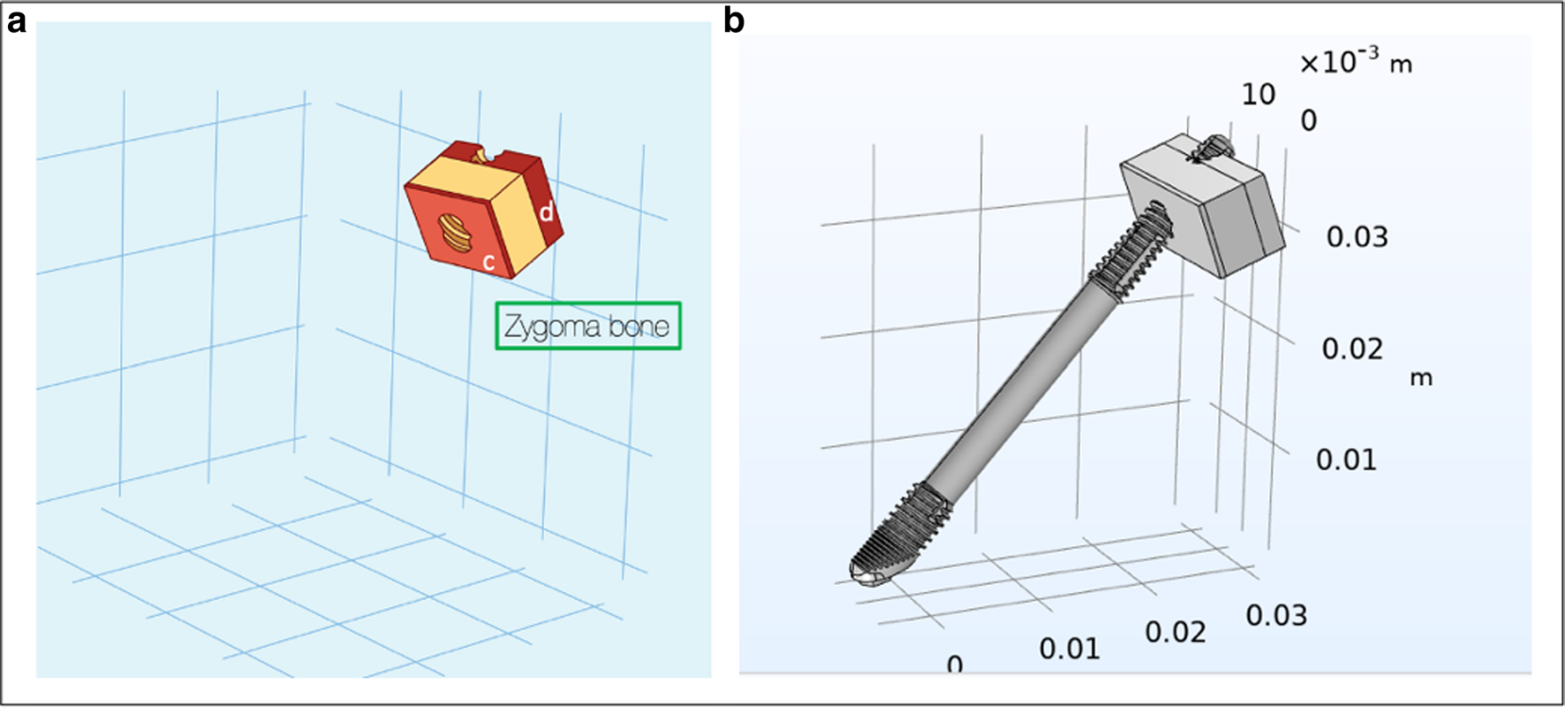
**a**, **b** Bi-cortical points for the zygoma implant stabilized only with in the zygoma bone

This refers to the zygoma implant platform having no boney support from the maxilla. The apex of the implant is the only point of bone to implant contact (BIC) stabilized by the cortical bone of the roof of the maxillary sinus as well as the lateral cortical wall of the zygoma bone, c and d as represented in Fig. 15a, b. Therefore, the implant has only bi-cortical stabilization.

As mentioned earlier, studying the transfer of occlusal forces to the zygoma implant, the residual maxillary crest as well as the zygoma bone is clinically relevant for treatment planning with the optimal biomechanical principles in mind.

The focus of the Bedrossian–Brunski study was to observe the effect of applying combined vertical load of 100 N as well as horizontal load of 50 N (representing mid-to-lower bite force) to the zygoma implant with Quad-cortical stabilization (QCS), as well as zygoma implant with bi-cortical stabilization (BCS). The magnitude of the applied forces to the QCS zygoma implants were measured and contrasted to the BCS zygoma implant model.

The following applied forces were measured:

- Vertical displacement of the zygoma implant.
- Applied combination of vertical and horizonal forces.

#### Vertical displacement

Vertical force of 10 Ncm was applied to the zygoma implant platform to measure the degree of vertical displacement both in the QCS and the BCS zygoma implant models.

**Fig. 16:**
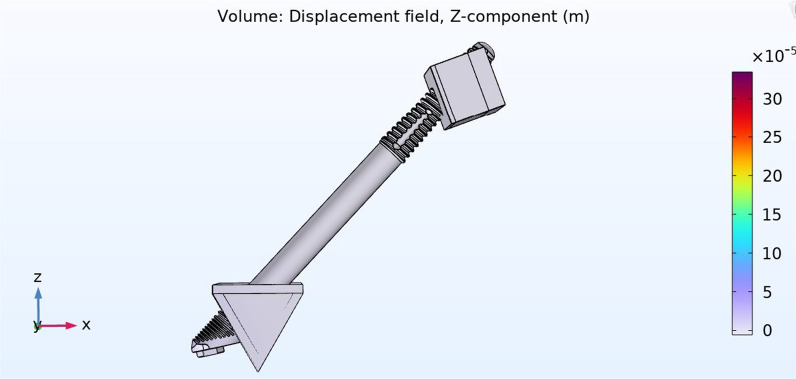
11 µm of vertical displacement on the quad-cortically stabilized zygoma implant

**Fig. 17:**
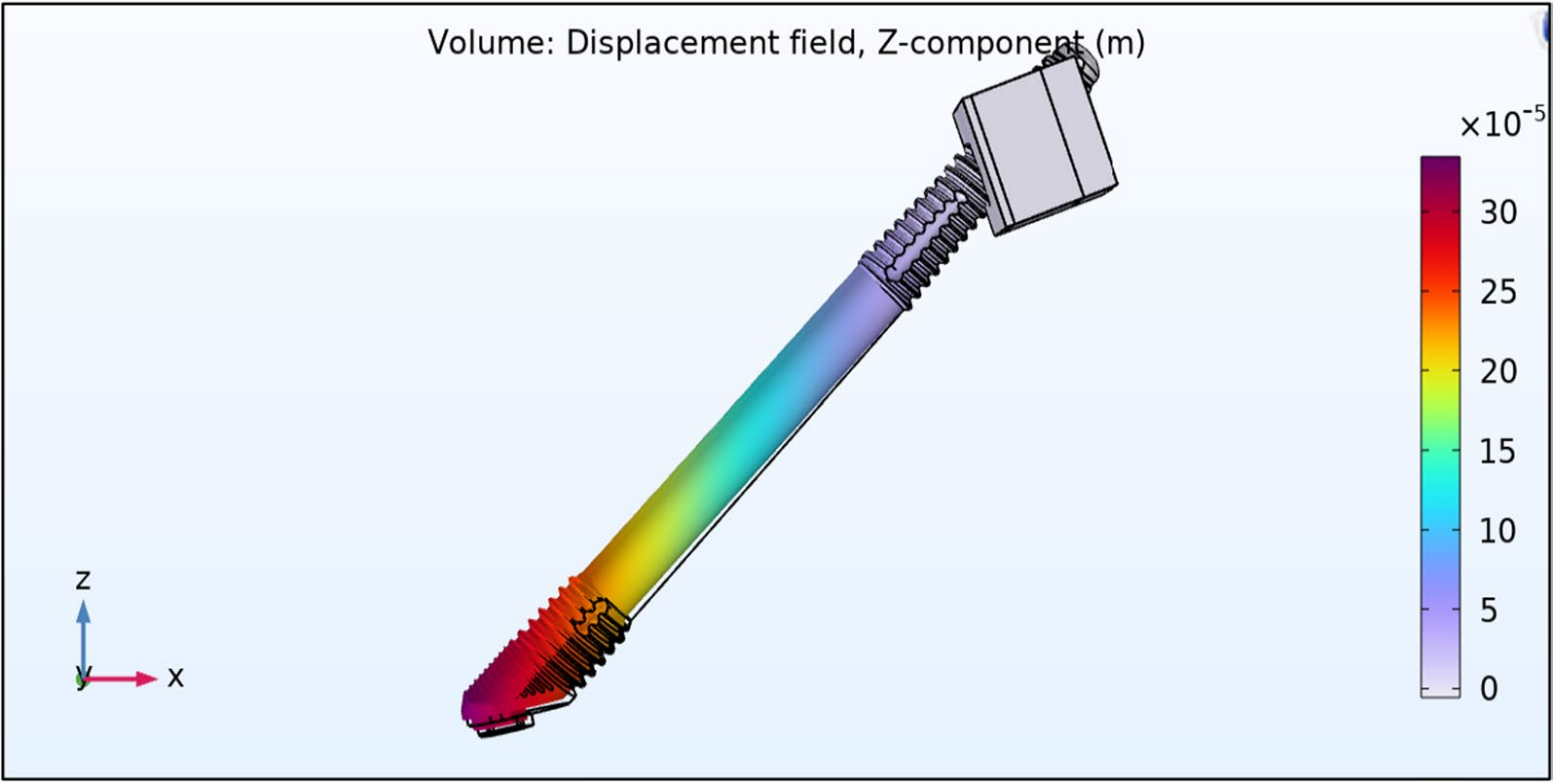
300 µm of vertical displacement on the bicortically stabilized zygoma implant

The vertical displacement of a QCS zygoma implant was 11 µm in the + *z*. axis with a vertical stiffness of approximately 10 N/11 µm or 0.909 N/µm (~ 1 N/µm) (Fig. 16). The vertical displacement of a BCS zygoma implant was 300 µm in the + *z*. axis with a vertical stiffness is approximately 10 N/300 µm =  ~ 0.03 N/µm (Fig. 17).

It is therefore clear that the residual maxilla contribute significantly in limiting the vertical displacement of the zygoma implant.

#### Applied combination of vertical and horizonal forces

**Fig. 18:**
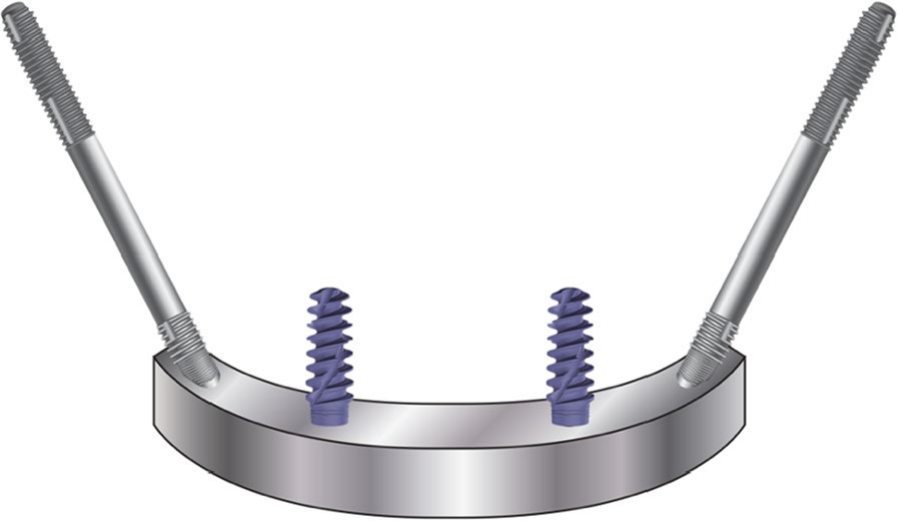
Cross-arch splinted zygoma implants with the premaxillary implants

The effects of combined 100 N vertical and 50 N horizontal loading of the zygoma implant were considered as this combined force application mimics closely the functional forces applied to the prosthesis by patients. In the splinted model (Fig. 18) the forces were calculated on each of the 4 implants using analytical models [21–23] that account for differences that can exist in the stiffness of each implant in the bone.

**Fig. 19:**
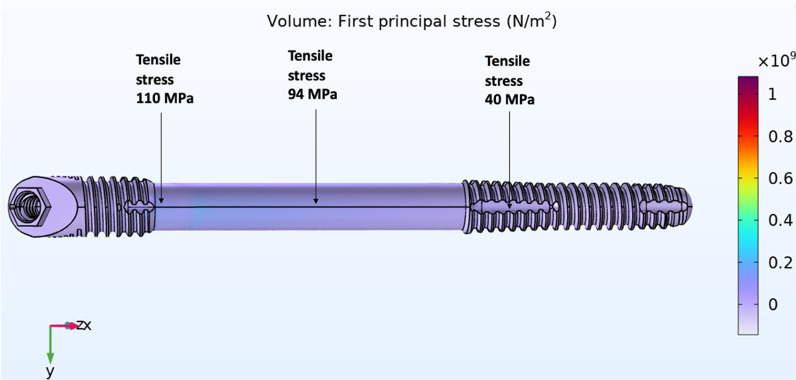
Quad-cortical, cross-arch splinted *z*-implant – *stresses in the implant* combined horizontal + vertical loading

**Fig. 20:**
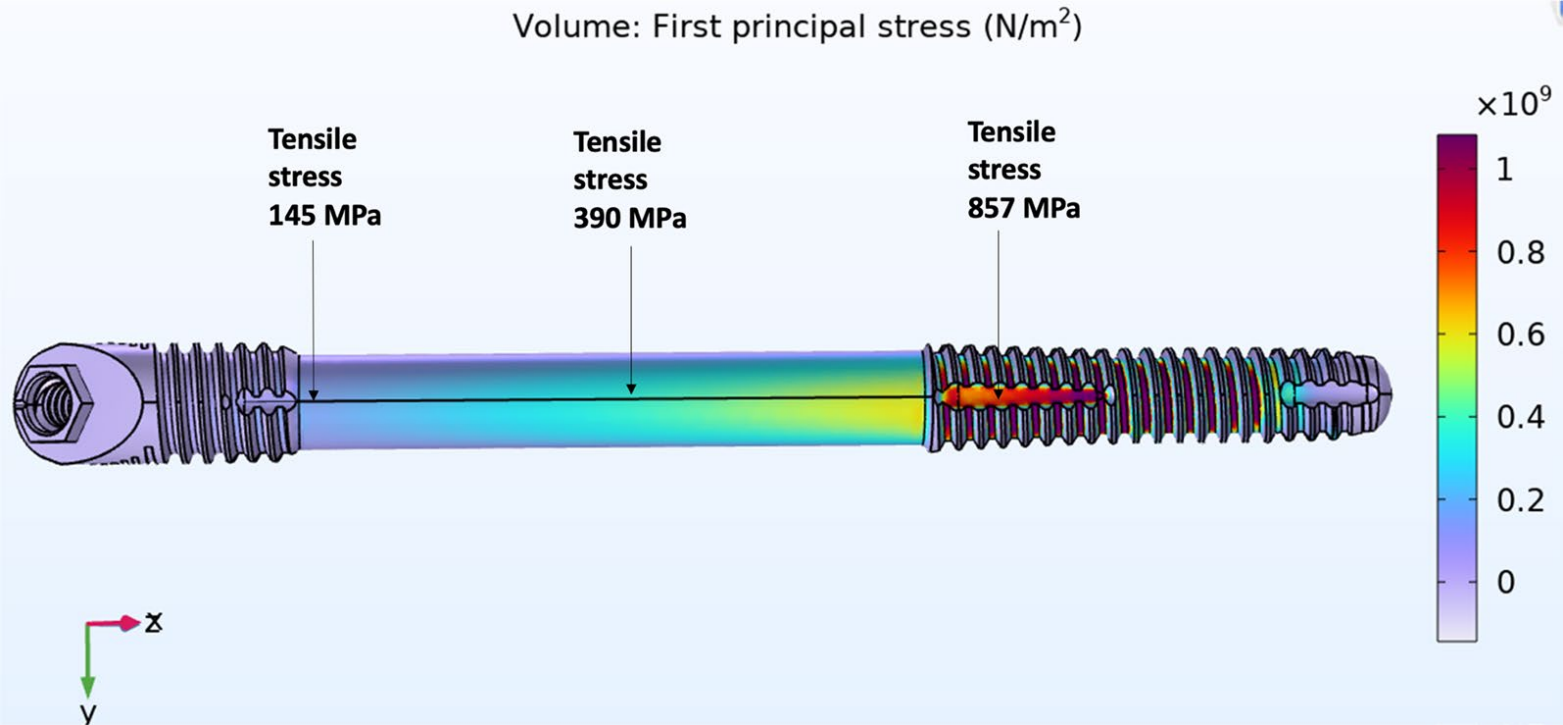
BICORTICAL, cross-arch splinted *z*-implant – *stresses in the implant.* Combined horizontal + vertical loading

The magnitude of tensile forces created in 3 areas of the zygoma implant; the implant platform area, mid-shaft and its apex in the zygoma bone was measured. The result of the stress applied to the quad-cortically stabilized and splinted zygoma implant is represented in Fig. 19. In contrast, the bicortically stabilized and splinted implant mode as seen in Fig. 20, demonstrated increased levels of tensile stress within the zygoma implant. The tensile stress within the apical portion of the splinted zygoma implants increases from 40 to 857 MPa for the QCZ model as compared to the BCZ model, respectively.

Table 1 represents the forces in all three points of the zygoma implants under combined vertical and horizontal loads in the cross-arch splinted model.

**Table 1:** Combination of vertical and horizontal force application on cross-arch splinted zygoma implant

Cross-arch splinted *z*-implant	Quad-cortical stabilized (MPa)	Bi-cortical stabilized (MPa)
Platform	110	145
Mid-shaft	94	390
Apex	40	857

**Fig. 21:**
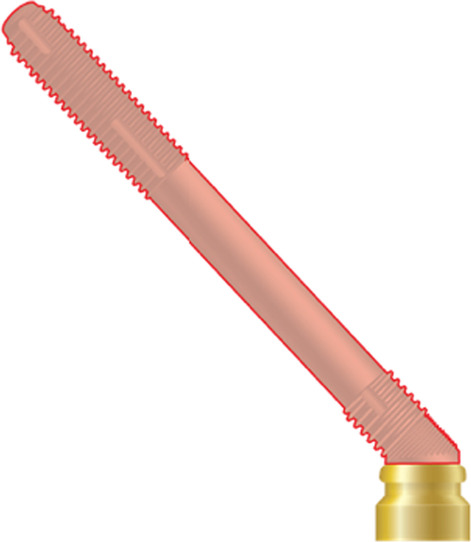
Lone standing zygoma implant

**Fig. 22:**
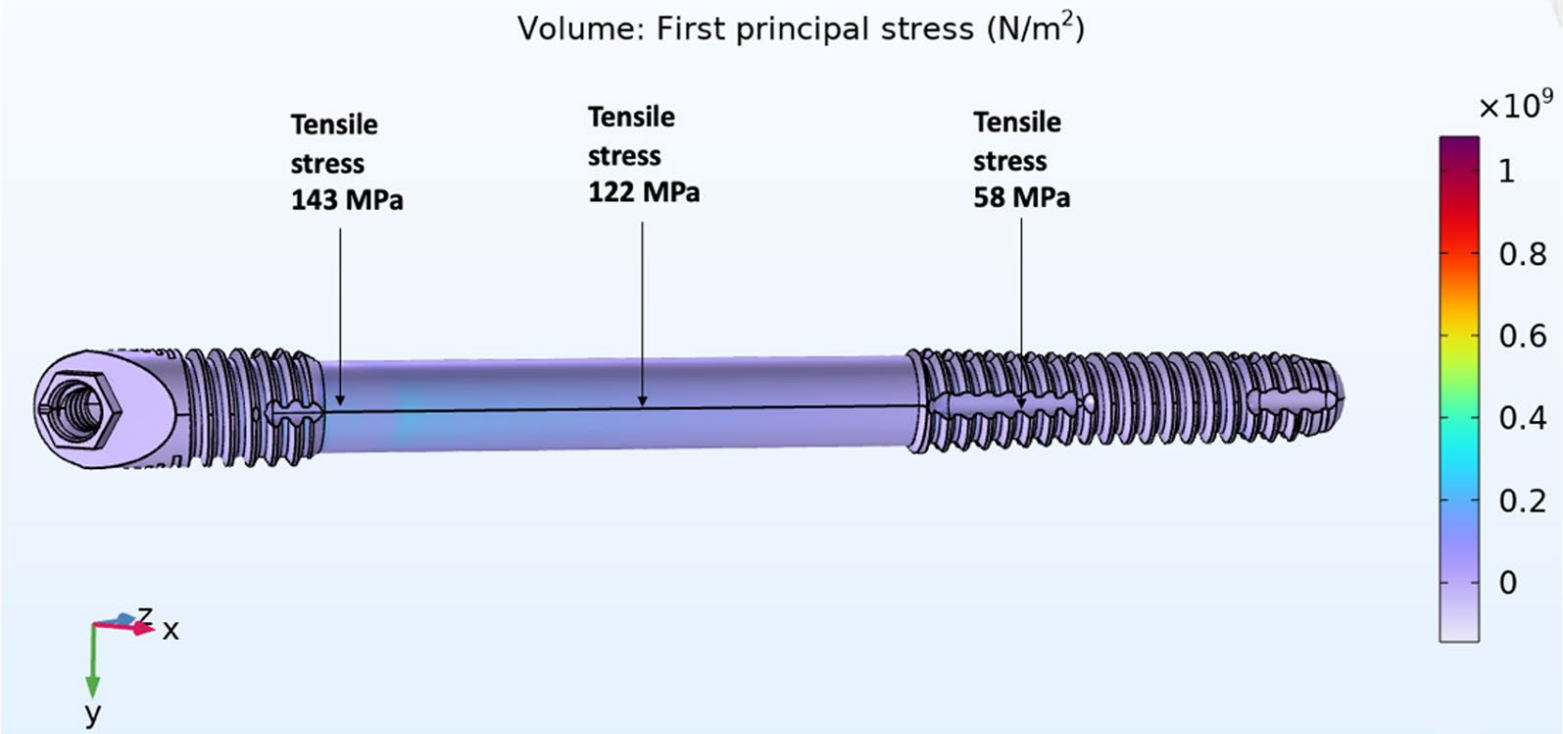
QUAD, free-standing *z*-implant – *stresses in the implant.* Combined horizontal + vertical loading

**Fig. 23:**
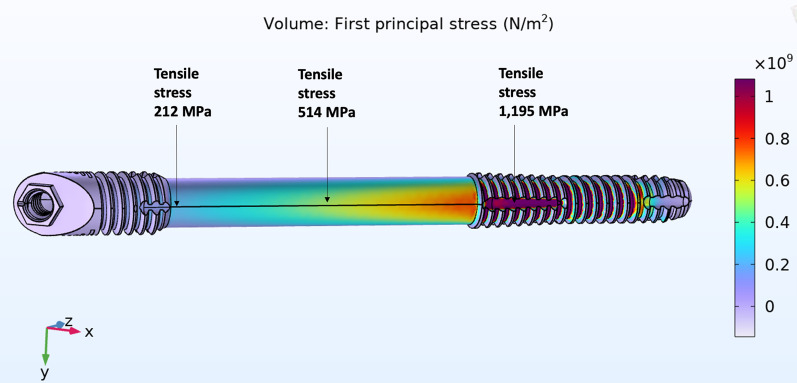
Bi-cortical, free-standing *z*-implant – *stresses in the implant.* Combined horizontal + vertical loading

For zygoma implants which were not cross-arch splinted (Fig. 21), the stresses measured within the zygoma implant when loaded with combined vertical and horizontal loads are represented in Figs. 22 and 23.

The magnitude of the stress at the apex of the quad-cortical, free-standing zygoma implant is 58 MPa in contrast to 1954 MPa for the free-standing bi-cortical zygoma implant in Fig. 26.

Table 2 represents the forces in all three points of the zygoma implants under combined vertical and horizontal loads in the free-standing model.

**Table 2:** Combination of vertical and horizontal force application on free-standing zygoma implants

Free-standing *z*-implant	Quad-cortical stabilized (MPa)	Bi-cortical stabilized (MPa)
Platform	143	212
Mid-shaft	122	514
Apex	58	1195

From the results outlined above, the stresses applied to the apical potion of the zygoma implant is increased from 40 to 847 MPa in the splinted quad-cortically stabilized vs the bicortically stabilized zygoma implant, respectively. What is more significant, is the increase in stress at the apical portion of the zygoma implant from 58 to 1195 MPa in the lone-standing quad-cortically stabilized vs the bicortically stabilized zygoma implants, respectively.

The data support the quad-cortical stabilization of the zygoma implant by the surgeon as well as the cross-arch splinting of the zygoma implants with the premaxillary implants by the restorative team is essential.

### Tensile forces within the maxillary and zygoma bone

**Fig. 24:**
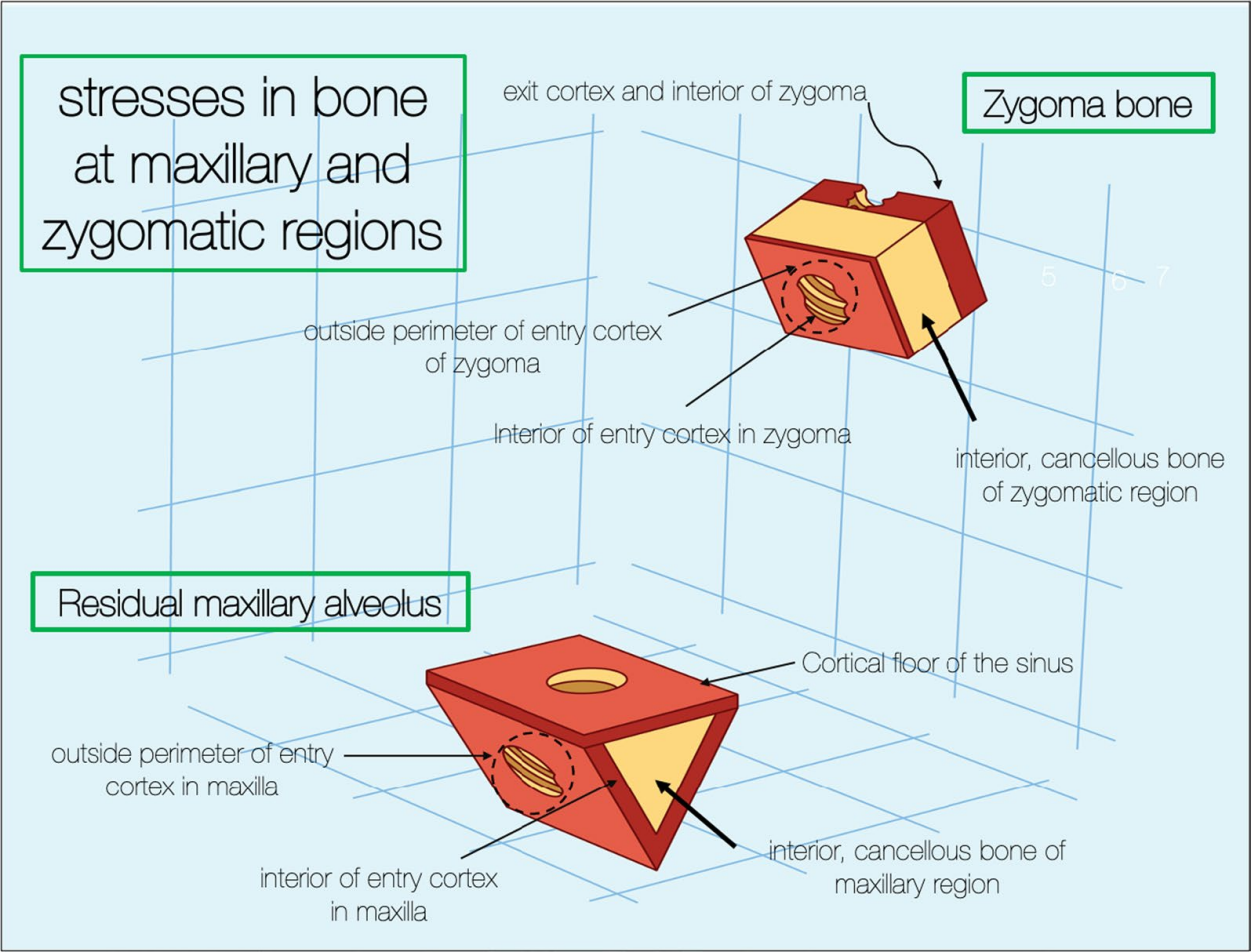
Points for the measurement of the stresses within maxilla and the zygoma bone

In order to evaluate the tensile stress within the residual maxillary alveolus as well as the zygoma bone using the BBQ and the BBB models, various measurements were assessed while applying a combined 100 N (vertical) and 50 N (horizontal) forces to the zygoma implant platforms. Figure 24 identifies the points of stress measurements for the residual maxillary ridge as well as the zygoma bone.

**Fig. 25:**
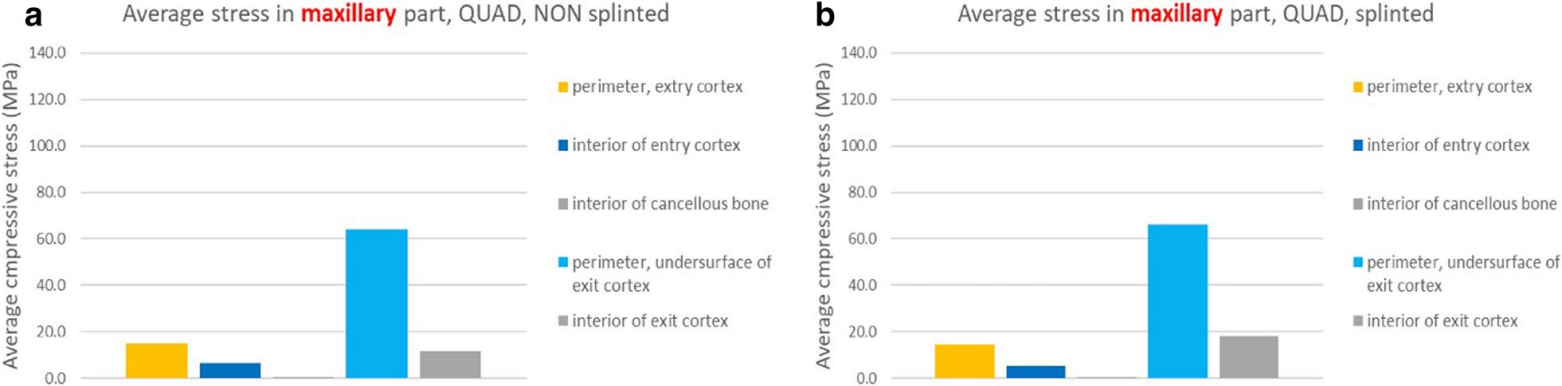
**a**, **b** BBQ non-splinted and splinted model showing the stress within the maxillary alveolar bone under functional loading

The tensile stresses created within the maxillary alveolar bone in the BBQ non-splinted and splinted models are presented in Fig. 25a, b. Under function loads, the maxillary lingual cortex, the interior cancellous bone of the alveolus as well as the cortical floor of the maxillary sinus floor support the stress produced under vertical and horizontal loading.

**Fig. 26:**
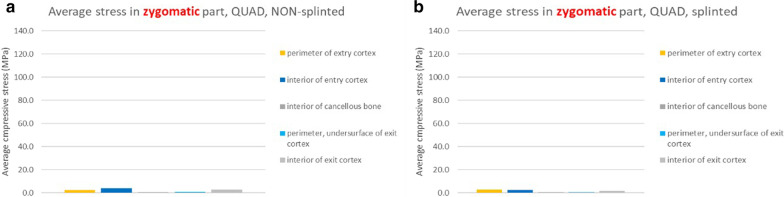
**a**, **b** BBQ non-splinted and splinted model showing the stress within the zygoma bone under functional loading

The tensile stresses created within the zygoma bone in the BBQ non-splinted and splinted models are presented in Fig. 26c, d. Under function loads, the quad-cortically stabilized zygoma implant results in nominal stress within the zygoma bone as the stresses are concentrated in the maxillary alveolus.

**Fig. 27:**
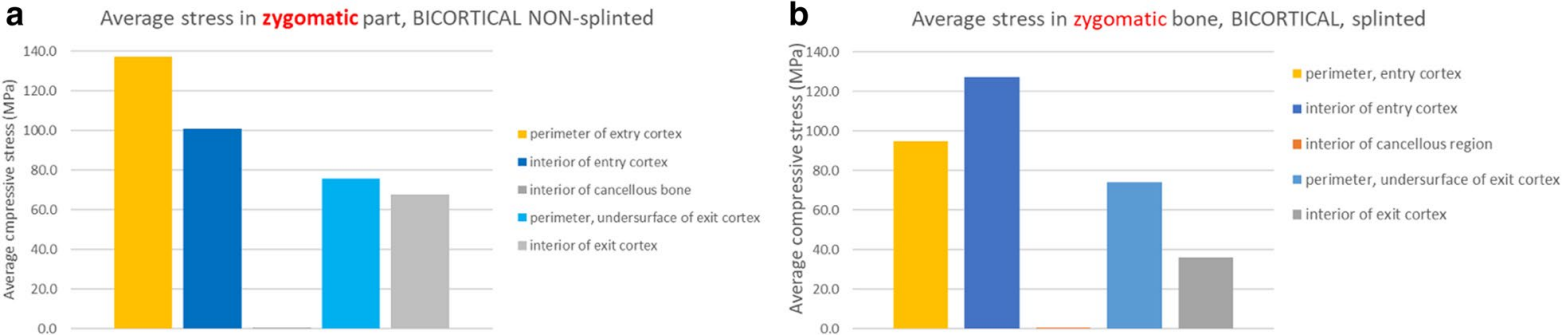
**a**, **b** BBB non-splinted and splinted model showing the stress within the maxillary alveolar bone under functional loading

The tensile stresses created within the zygoma bone in the BBB non-splinted and splinted models are presented in Fig. 27a, b. Under functional loads, without stabilization of the zygoma implant platform, increased tensile stresses are measured within the zygoma bone reaching levels of titanium and bone fatigue.

The measurements of stress in the zygoma bone both on the cortical and the cancellous components, clearly illustrates the increased magnitude of stress in cases where the zygoma implant is only stabilized at its apex (bicortically) within the zygoma bone. The stabilization of the zygoma implant within the residual maxillary crestal bone significantly reduces the degree of stress applied to the zygoma bone in the quad-cortically stabilized zygoma implant.

The biomechanical evidence to date confirms that the quad-cortical stabilization of the zygoma implant is desirable and possible in ZAGA 0, 1, 2 and 3 cases. The authors are also cognizant of the potential resorption of the 2–3 mm of the maxillary alveolar bone which was engaged initially at the time of implant placement. It is very rare to have the opportunity to surgically inspect the presence or the lack of the alveolar bone in subsequent follow-up visits. Therefore, the potential for the partial vs total resorption of the alveolar bone although reasonable to consider, is hypostasized and not proven. The intentional removal of the maxillary crestal bone is discouraged and engaging the maxillary crestal bone in ZAGA 0, 1, 2 and 3 cases is strongly recommended.

In 2023, Varghese and colleagues from the Department of Prosthodontics at the Christian Dental College, Ludhiana, Punjab, India, published a biomechanical study entitled, *“Three-dimensional finite element analysis of zygomatic implants for rehabilitation of patients with a severely atrophic maxilla”* in JPD [24]. Their article begins with:

#### Statement of problem

Stresses applied to zygomatic implants have been determined to be transferred mainly to the zygomatic bone; however, consensus regarding the stress distribution pattern in the bone surrounding zygomatic implants has not yet been reached.

There review of the published articles over the last two decades as well as their FEA reached the same results as reported by Bedrossian and Brunski with the BBQ and the BBB models. The conclusions of Varghese’s research were*:*

“This result led to the rejection of the research hypothesis and was consistent with that of Freedman et al, challenges the widely held belief that stresses from the zygomatic implants are primarily dissipated through the zygomatic bone.”

With clarification of the literature, understanding and adopting the most favorable biomechanical principles using the zygoma implant is possible. Therefore, it is prudent to review the treatment planning protocol utilizing this treatment concept.

### Treatment planning using the zygoma implant

**Fig. 28:**
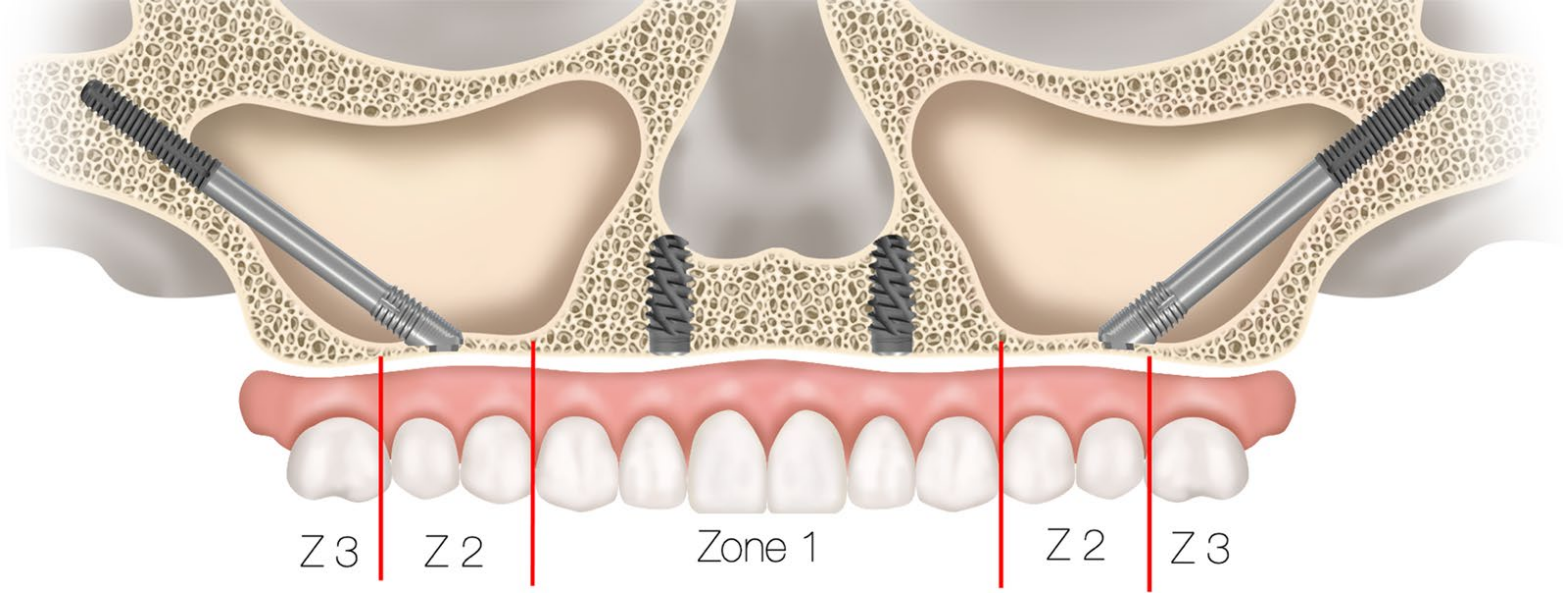
Zones of the maxilla determine the indication for the zygoma concept

To determine whether a patient who presents with resorbed maxilla is a candidate for zygoma implants, 2-dimensional as well as 3-dimensional radiographic evaluation techniques are most useful. The use of the 2-dimensional radiographic evaluation was described by Bedrossian [25]. To efficiently evaluate whether the patient is a candidate for treatment with the zygoma concept, identification of the presence or the lack of the maxillary ZONES is considered. The maxillary arch is divided in to 3 ZONES. Zone 1 being the premaxilla, ZONE 2, the bicuspid space and ZONE 3 the molar space. The lack of bone in Zones 2 and 3 are the determinants for placement of zygoma implants in the posterior maxilla in conjunction with 2 or 4 axial implants in Zone 1, the premaxilla (Fig. 28).

**Fig. 29:**
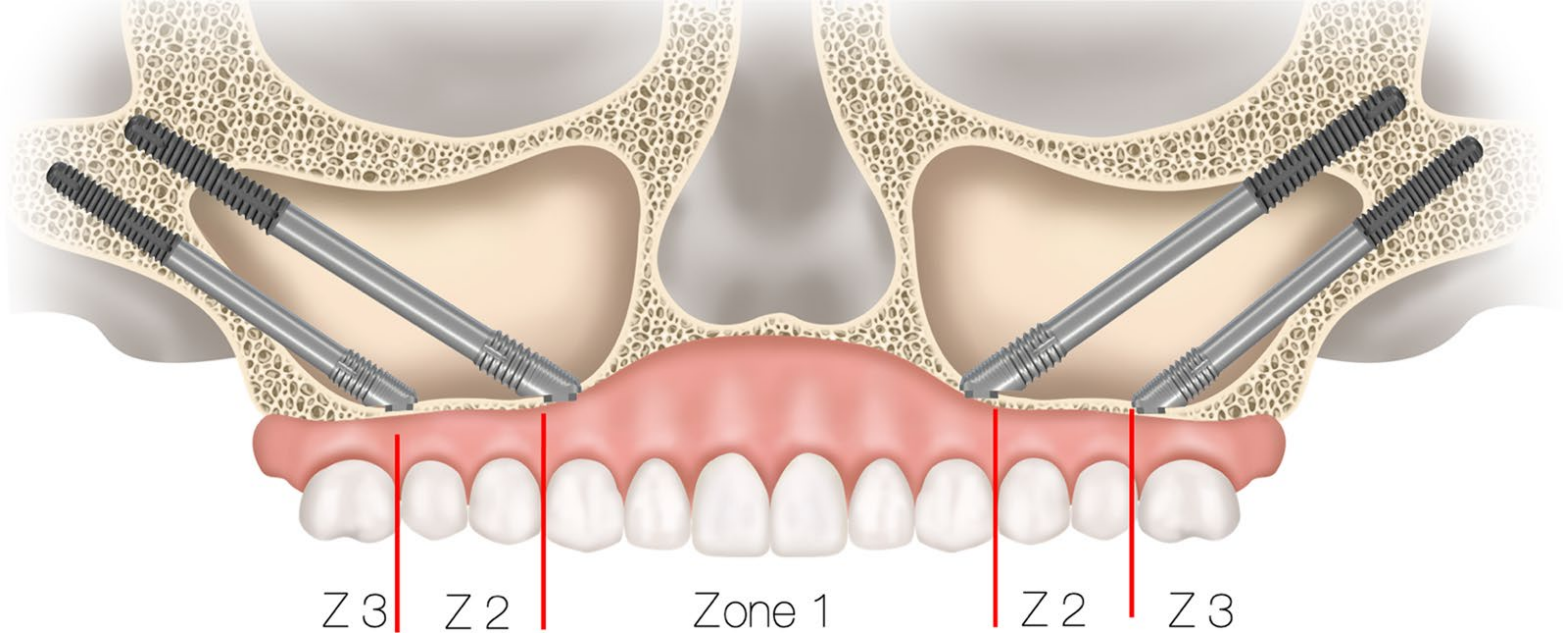
Zones of the maxilla determines the indication for the quad-zygoma concept

In cases where total maxillary alveolar atrophy is observed, with bilateral lack of bone in Zones 1, 2 and 3, the quad-zygoma concept is considered (Fig. 29).

**Fig. 30:**
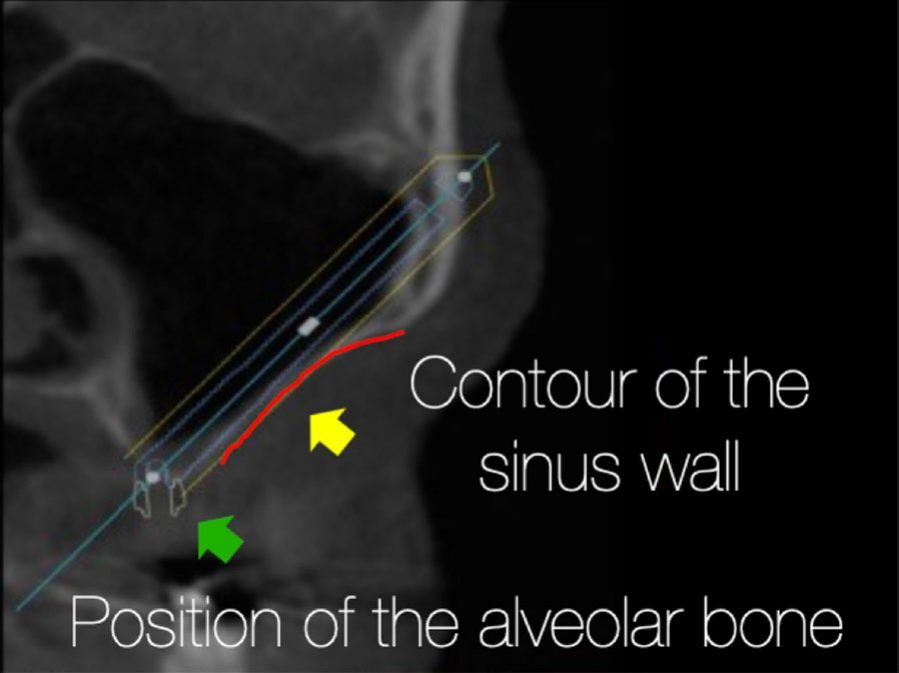
ZAGA classification, describes the contour of the lateral sinus wall and the position of the residual maxillary crest

Once the use of the zygoma implant is determined to be the treatment of choice, the 3-dimensional radiographic study as described by Aparicio [26], is considered. The zygoma anatomy guided approach, ZAGA classification, describes the degree of concavity of the lateral maxillary sinus wall as well as the degree of palatal resorption of the residual maxillary crest (Fig. 30).

**Fig. 31:**
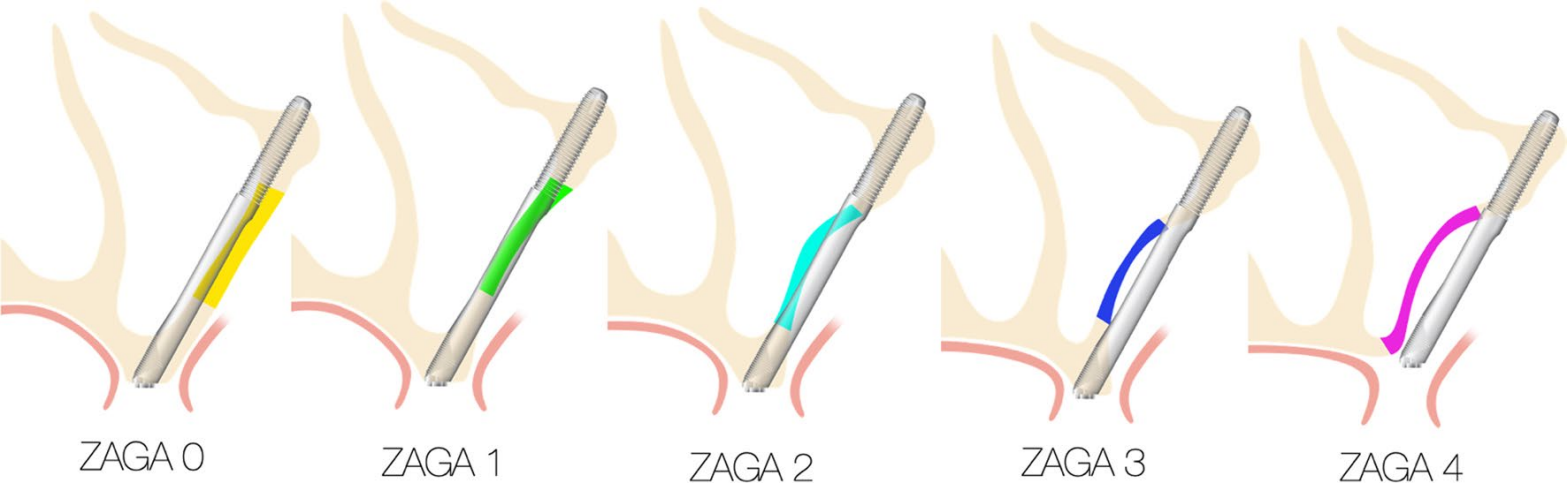
The zygomatic anatomic classification

The ZAGA classification ranges from 0 to 4. The clinician may import the patient’s 3-D scan into a planning software of their choice and positions a simulated zygoma implant with the appropriate trajectory as described by the OST. By doing so, the clinician can visualize and predict whether the platform of the zygoma implant will be stabilized in the patient’s premaxilla as well as whether the mid portion of the zygoma implant will be completely inside, partially inside or completely outside the maxillary sinus (Fig. 31).

**Fig. 32:**
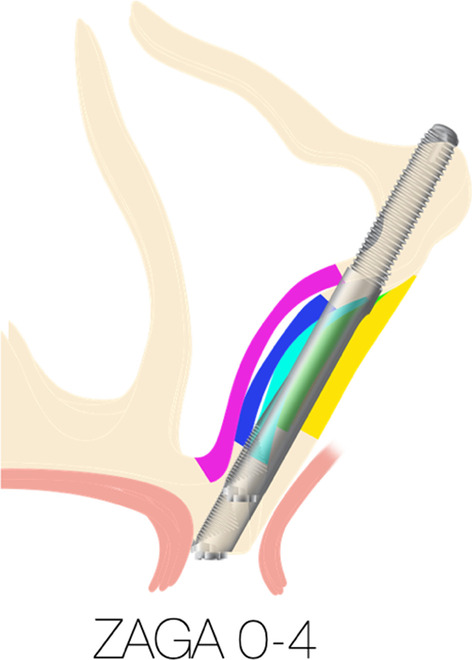
Superimposition of the of ZAGA 0 to ZAGA 4, the various levels of sinus wall concavity is illustrated by different colors

As reported by Aparicio, 93.8% of the patients studied had anatomic presentation between ZAGA 0–3 and only 6.5% of the patients had topography consistent with ZAGA 4. Therefore, quad-cortical stabilization should be possible in most cases. Figure 32 is an illustration of the superimposed ZAGO 0 to ZAGA 4. The implant platform, the implant apex and the implant trajectories directly superimpose on top of each other. The only variable is the concavity of the lateral maxillary sinus wall as well as the palatal resorption of the maxillary alveolus. This resorption pattern does not allow the stabilization of the implant platform in bone for ZAGA 4 cases.

It is also important to discuss the contour of the fixed prosthesis, (FP), supported by zygoma implants. Some clinicians incorrectly refer to the zygoma implants being placed “too palatal” resulting in the screw access holes of the FP to also be “too palatal”. To better understand the nuances of the FP supported by zygoma implants, the palatal resorption pattern of the maxillary alveolar bone requires discussing.

**Fig. 33:**
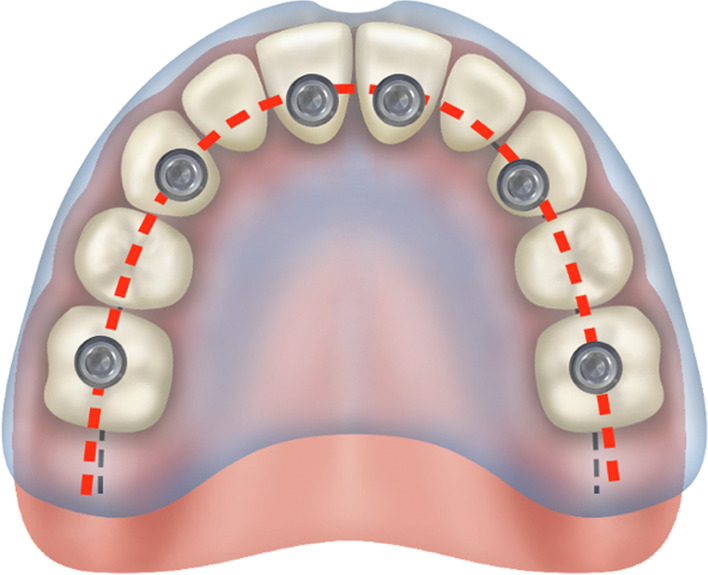
Non-resorbed maxilla with the arch form, red-dotted line. Superimposed on the arch form, the black-dotted line

In the non-resorbed edentulous maxilla, the crest of the edentulous maxillary alveolus is represented by the “black-dotted line”. The arch form is represented by the “red-dotted line”. In the non-resorbed maxilla, the red-dotted line would be superimposed on top of the black-dotted line placing the screw access holes in the cingulum and the central fossa of the anterior and posterior teeth of the FP, respectively (Fig. 33).

**Fig. 34:**
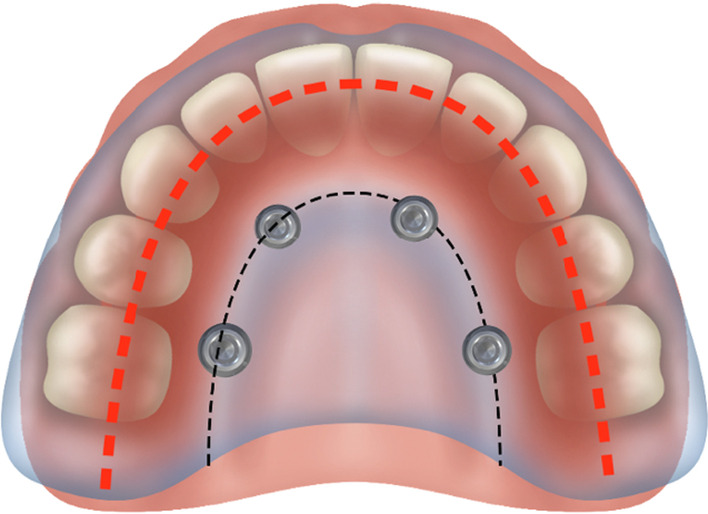
Placement of the zygoma implant platform on the resorbed residual ridge with the prosthetic access holes palatal to red-dotted line

However, with the palatal resorption pattern of the maxillary alveolus, the black-dotted line is palatal to the red-dotted line representing the patient’s arch form. With the implants placed over the resorbed ridge, the screw access holes emerge more palatal as compared to none-resorbed maxilla as seen in Fig. 34 [27].

It is important for the implant team to appreciate that with the palatal resorption of the maxillary alveolar bone, the removal of the maxillary crestal alveolus as described by the “extra-sinus technique” will not result in any better positioning of the zygoma implant platform. In isolated cases where the edentulous maxilla resorbs mostly upward with minimal palatal resorption, the screw access hole may be positioned as seen in the non-resorbed maxilla.

By appreciating the anatomic limitations presented with patients suffering from moderate to severe maxillary resorption, providing crestal mechanical anchorage for the zygoma implant will optimize the force distribution of the FP.

## Conclusion

By following the aforementioned biomechanical principles, reconstruction of the atrophic maxillae with the zygoma concept is very predictable as reported in systematic reviews by Chrcanovic and Goiato [28, 29].

The maxillary alveolus is the primary support for zygoma implants under function and therefore it is recommended, when possible, to stabilize the zygoma implant both at the maxillary alveolar crest as well as the zygoma bone at time of placement. Care should be taken to preserve the crestal alveolus to allow bi-cortical stabilization of the zygoma implant platform. The provisional prosthesis as well as the final prosthesis, should always cross-arch splint the posterior zygoma implants with the premaxillary implants for favorable force distribution under functional loads. The loading of the non-splinted zygoma implant is not recommended.

## Data Availability

The authors have reviewed the data existing in the current literature and have conducted their FEA study with reporting of the data in this document.

## Abbreviations

ZAGA: Zygoma anatomy guided approach
CT: Computer tomography
FEA: Finite element analysis
BIC: Bone-to-implant contact
QCS: Quad-cortical stabilization
BCS: Bi-cortical stabilization
BBQ: Bedrossian Brunski quad-cortical
BBB: Bedrossian Brunski bi-cortical
FP: Fixed prosthesis

## References

[1] Brånemark PI, Gröndahl K, Ohrnell LO, Nilsson P, Peterson B, Svensson B, Engstrand P, Nannmark U (2004). Zygoma fixture in the management of advanced atrophy of the maxilla: technique and long-term results. Scand J Plast Reconstr Surg Hand Surg.

[2] Bedrossian E, Rangers B, Stumpel L, Indersano T (2006). Immediate function with the zygomatic implant: a graftless solution for the patient with mild to advanced atrophy of the maxilla. Int J Oral Maxillofac Implants.

[3] Chow J, Hui E, Lee PK (2006). Zygomatic implants—protocol for immediate occlusal loading: a preliminary report. J Oral Maxillofac Surg.

[4] Branemark PI. Surgery and fixture installation: zygomaticus fixture clinical procedures. Gothenburg, Sweden, Nobel Biocare AB; 1998, pp. 1.

[5] Petruson B (2004). Sinuscopy in patients with titanium implants in the nose and sinus. Scand J Plast Reconstr Surg Hand Surg.

[6] Malo P (2008). A new approach to rehabilitate the severely atrophic maxilla using extramaxillary anchored implants in immediate function: a pilot study. J Prosthet Dent.

[7] Pierrisnard L, Renouard F, Renault P, Barquins M (2003). Influence of implant length and bicortical anchorage on implant stress distribution. Clin Implant Dent Relat Res.

[8] Meijer HJ, Kuiper JH, Starmans FJ, Bosman F (1992). Stress distribution around dental implants: influence of superstructure, length of implants, and height of mandible. J Prosthet Dent.

[9] Rieger MR, Mayberry M, Brose MO (1990). Finite element analysis of six endosseous implants. J Prosthet Dent.

[10] Bidez MW, Misch CE (1992). Issues in bone mechanics related to oral implants. Implant Dent.

[11] Kitamura E, Stegaroiu R, Nomura S, Miyakawa O (2004). Biomechanical aspects of marginal bone resorption around osseointegrated implants: considerations based on a three-dimensional finite element analysis. Clin Oral Implants Res.

[12] Iplikcioglu H, Akca K (2002). Comparative evaluation of the effect of diameter, length and number of implants supporting three-unit fixed partial prostheses on stress distribution in the bone. J Dent.

[13] Nishihara K, Nakagiri S (1994). Biomechanical studies on newly tailored artificial dental root. Biomed Mater Eng.

[14] Hedia HS (2002). Stress and strain distribution behavior in the bone due to the effect of cancellous bone, dental implant material and the bone height. Biomed Mater Eng.

[15] Rangert B, Krogh PH, Langer B, Van Roekel N (1995). Bending overload and implant fracture: a retrospective clinical analysis. Int J Oral Maxillofac Implants.

[16] Rangert B (1993). Mechanical and biomechanical guidelines for the use of Branemark system—general principles. Aust Prosthodont J.

[17] Kato Y, Kizu Y, Tonogi M, Ide Y, Yamane G-Y (2005). Internal structure of zygomatic bone related to zygomatic fixture. J Oral Maxillofac Surg.

[18] Freedman M, Ring M, Stassen LFA (2013). Effect of alveolar bone support on zygomatic implants: a finite element analysis study. Int J Oral Maxillofac Surg.

[19] Freedman M, Ring M, Stassen LFA (2015). Effect of alveolar bone support on zygomatic implants in an extra-sinus position—a finite element analysis study. Int J Oral Maxillofac Surg.

[20] Ujigawa K (2007). Three-dimensional finite elemental analysis of zygomatic implants in craniofacial structures. Int J Oral Maxillofac Surg.

[21] Skalak R, Brunski JB, Mendelson M. A method for calculating the distribution of vertical forces among variable-stiffness abutments supporting a dental prosthesis. In: Langrana NA, Friedman MH, Grood ES, editors. Bioengineering Conference, BED-Vol. 24, ASME, NY, 1993; pp. 347–350.

[22] Skalak R (1983). Biomechanical considerations in osseointegrated prostheses. J Prosthet Dent.

[23] Morgan MJ, James DF (1995). Force and moment distributions among osseointegrated dental implants. J Biomech.

[24] Varghese KG (2023). Three-dimensional finite element analysis of zygomatic implants for rehabilitation of patients with a severely atrophic maxilla. J Prosthet Dent.

[25] Bedrossian E (2008). Fixed prosthetic implant restoration of the edentulous maxilla: a systematic pretreatment evaluation method. J Oral Maxillofac Surg.

[26] Aparicio C (2011). A proposed classification for zygomatic implant patients based on the zygoma anatomy guided approach (ZAGA): a cross-sectional survey. Eur J Oral Implantol.

[27] Bedrossian E, Bedrossian EA (2019). Fundamental principles for immediate implant stability and loading. Compendium.

[28] Chrcanovic BR (2013). Survival and complications of zygomatic implants: a systematic review. Oral Maxillofac Surg.

[29] Goiato MC (2014). Implants in the zygomatic bone for maxillary prosthetic rehabilitation: a systematic review. Int J Oral Maxillofac Surg.

